# The postcranial anatomy of *Gorgonops torvus* (Synapsida, Gorgonopsia) from the late Permian of South Africa

**DOI:** 10.7717/peerj.15378

**Published:** 2023-07-07

**Authors:** Eva-Maria Bendel, Christian F. Kammerer, Roger M. H. Smith, Jörg Fröbisch

**Affiliations:** 1 Institut für Biologie, Humboldt Universität zu Berlin, Berlin, Germany; 2 Museum für Naturkunde, Leibniz-Institut für Evolutions- und Biodiversitätsforschung, Berlin, Germany; 3 Evolutionary Studies Institute, University of the Witwatersrand, Johannesburg, South Africa; 4 North Carolina Museum of Natural Sciences, Raleigh, North Carolina, United States of America; 5 Department of Karoo Palaeontology, Iziko South African Museum, Cape Town, South Africa; 6 Leibniz-Institut für Evolutions- und Biodiversitätsforschung, Museum für Naturkunde, Berlin, Germany

**Keywords:** Synapsida, Therapsida, Gorgonopsia, Permian, Taxonomy, Gorgonops torvus, Postcranial anatomy

## Abstract

Gorgonopsians are among the most recognizable groups of synapsids from the Permian period and have an extensive but mostly cranial fossil record. By contrast, relatively little is known about their postcranial anatomy. Here, we describe a nearly complete, semi-articulated skeleton of a gorgonopsian (identified as *Gorgonops torvus*) from the late Permian *Endothiodon* Assemblage Zone of the South African Karoo Basin and discuss its paleobiological implications. Known gorgonopsian postcrania indicate morphological conservatism in the group, but the skeletal anatomy of *Gorgonops* does differ from that of other gorgonopsians in some respects, such as in the triangular radiale and short terminal phalanges in the manus, and a weakly developed distinction between pubis and ischium in ventral aspect of the pelvic girdle. Similarities between the specimen described herein and a historically problematic specimen originally referred to “*Scymnognathus* cf. *whaitsi*” confirm referral of the latter specimen to *Gorgonops*. Since descriptions of gorgonopsian postcrania are rare, new interpretations of the lifestyle and ecology of Gorgonopsia can be drawn from our contribution. We conclude that gorgonopsians were likely ambush predators, able to chase their prey over short distances and pin them down with strong forelimbs before using their canines for the kill. This is evidenced by their different fore- and hindlimb morphology; the former stouter and more robust in comparison to the longer, more gracile, back legs. Furthermore, the completeness of the study specimen facilitates calculation of an estimated body mass of approximately 98 kg, similar to that of a modern lioness.

## Introduction

In the late 19^th^ and early 20^th^ centuries, gorgonopsian cranial anatomy was the focus of a plethora of publications, most involving descriptions of new taxa (*e.g*., Boonstra, 1934a, 1934b, 1965; Boonstra & Broom, 1935; Broom, 1913a, 1913b, 1930, 1935a, 1935b; Owen, 1881). However, many of these descriptions were somewhat cursory, being based upon vague and sometimes contradictory characters, thus complicating later studies of these taxa. In the following decades there were only a few published descriptions of gorgonopsians specifically as opposed to therapsids generally (*e.g*., Colbert, 1948; Kemp, 1969a, 1969b; Sigogneau, 1970, 1974; Sigogneau-Russell, 1989). Recently there has been renewed scientific interest in gorgonopsian anatomy, but thus far the primary focus has remained cranial (Araújo et al., 2017; Bendel et al., 2018; Kammerer, 2014, 2015, 2016, 2017a, 2017b; Kammerer & Masyutin, 2018; Kammerer et al., 2015).

While work on gorgonopsian skulls is experiencing a renaissance, our knowledge of gorgonopsian postcranial anatomy has remained minimal. In part due to a historic worker bias for only collecting skulls and taphonomic biases favoring skull preservation in the most heavily-sampled gorgonopsian-bearing strata (*e.g*., the Beaufort Group of South Africa; see Smith (1993)), gorgonopsian postcrania are rare in museum collections, and few descriptions of them exist. The earliest entry in the gorgonopsian postcranial literature is that of Owen (1876a), who described an isolated humerus that he referred to the taxon *Cynodraco major* (currently considered a *nomen dubium* (Sigogneau-Russell, 1989), but, contra Sigogneau (1970), demonstrably gorgonopsian). Broom & Haughton (1913) were the first to describe an associated gorgonopsian skeleton, including the skull, vertebrae, shoulder girdle, and forelimb of the specimen SAM-PK-2342 (holotype of their new taxon *Scymnognathus tigriceps*; currently *Aelurognathus tigriceps* (Kammerer, 2016)). Subsequent publications, such as those of Broom (1915) and Watson (1917), were less focused on the description of particular gorgonopsian specimens and more about using them as exemplars in reconstructing evolutionary changes leading to mammals. The first nearly complete skeletons of gorgonopsians, representing the giant Russian taxon *Inostrancevia*, were actually discovered in the late 19^th^ century, but due to protracted preparation and the death of the lead researcher (V. P. Amalitzky), details of their anatomy were not published until decades later (Pravoslavlev, 1927b).

The most detailed historic descriptions of gorgonopsian postcrania were published between 1930 and 1960. Boonstra (1934a) recognized the paucity of postcranial specimens and descriptions for the group, and following a review of the (limited) literature described several specimens based on semi-articulated materials, notably a partial skeleton (SAM-PK-9344) that he made the holotype of the new species *Aelurognathus microdon* (*Lycaenops*? *microdon* of Sigogneau (1970)). Broili & Schröder (1935) described a specimen (BSPG 1934 VIII 28) that they referred to the genus *Scymnognathus*, albeit of uncertain specific attribution (*S*. cf. *whaitsi*). The identification of this specimen has remained vague in more recent work, with Sigogneau (1970) listing it as *Gorgonops* cf. *whaitsi* and Gebauer (2007) considering it only as Gorgonopsia indet. Taxonomic difficulties aside, the Broili & Schröder (1935) description represented an important step forward for research on African gorgonopsians, including for the first time a complete reconstruction of the skeleton showing an upright posture. This reconstruction has been debated in later articles on the skeletal posture of gorgonopsians, with Kemp (1982) arguing for more sprawling forelimbs and Gebauer (2007) maintaining that at least a facultatively erect gait for the forelimbs would have been possible.

Colbert (1948)’s monograph on the holotype of *Lycaenops ornatus* (AMNH FARB 2240) remains the most thorough description of any single gorgonopsian specimen. Covering the entire skeleton, this work is also remarkable (for the time) for including detailed comparisons with other gorgonopsian materials, including BSPG 1934 VIII 28, SAM-PK-2342, and SAM-PK-9344. Shortly thereafter, von Huene (1950) described another largely complete gorgonopsian skeleton, IGP U28 (later numbered GPIT/RE/7113 and currently GPIT-PV-31579), the holotype of *Scymnognathus parringtoni* (later reclassified as *Aelurognathus*? *parringtoni* by Sigogneau (1970) and *Sauroctonus parringtoni* by Gebauer (2014)).

More recent work on gorgonopsian postcrania has been sparse, but a few contributions are noteworthy. Boonstra (*e.g*., Boonstra, 1934b, 1965) described limb and girdle elements from the middle Permian *Tapinocephalus* Assemblage Zone of South Africa, although most of these are now considered to belong to biarmosuchians rather than gorgonopsians (Sigogneau-Russell, 1989). Kemp (1969a) compared the atlas-axis complex in various therapsids, including gorgonopsians, using information from several Tanzanian specimens (the “*Scymnognathus*” *parringtoni* type and two otherwise undescribed gorgonopsians in the collections of the University Museum of Zoology, Cambridge). Sigogneau-Russell (1989) compiled the known literature on gorgonopsian postcrania, figuring the majority of known gorgonopsian postcranial elements, but did not make explicit comparisons between taxa. Gebauer (2014) redescribed the holotype of “*Scymnognathus*” *parringtoni*, reassigning it to the Russian genus *Sauroctonus*. Tatarinov (2004) described the most complete gorgonopsian skeleton yet found (PIN 2212/61, the holotype of *Viatkogorgon ivakhnenkoi*), an articulated skeleton that includes even rarely preserved skeletal elements such as the gastralia and sclerotic ossicles. And most recently, Sidor (2022) revisited gorgonopsian pedal morphology on the basis of some exceptionally preserved specimens from Zambia (albeit unidentified in the absence of cranial material).

Though all of the above are valuable contributions to our knowledge on gorgonopsian postcranial anatomy, they still cover only a small fraction of the currently recognized gorgonopsian species, even after substantial reduction in number due to taxonomic revision (see *e.g*., Kammerer, 2015, 2016, 2017a). This is highly problematic for understanding gorgonopsian paleobiology and their distribution (geographic and temporal). Given that all current gorgonopsian species diagnoses are based solely on cranial characters, the postcranial record is currently completely excluded from range charts and tallies of species abundance. The potential importance of postcranial anatomy for taxonomic differentiation is also largely unknown; although gorgonopsian crania are very conservative in morphology, and known skeletons seem to show a comparable degree of homomorphism (Kemp, 1969b), this still remains to be tested in detail.

Here, we describe a recently collected specimen (SAM-PK-K10591) that we refer to the ‘archetypical’ gorgonopsian, *Gorgonops torvus*. Its near completeness offers valuable insights into gorgonopsian anatomy, allowing us to document details rarely seen in gorgonopsians, such as an articulated manus and pes. Our investigations of gorgonopsian postcrania confirm a stark conservatism in known specimens, and the variation and potential for distinction between taxa is therefore limited. Even so, some notable variation between taxa is observed in certain elements. Moreover, our results support reassignment of the historically dubious specimen BSPG 1934 VIII 28 to *Gorgonops*, and support some new paleobiological inferences on the lifestyle of this taxon.

**Systematic Paleontology**

Synapsida Osborn, 1903

Therapsida Broom, 1905

Gorgonopsia Seeley, 1894

*Gorgonops torvus* Owen, 1876b

**Revised Diagnosis**

The genus *Gorgonops* has yet to be revised in a modern taxonomic context. The most recent published account is that of Sigogneau-Russell (1989), who tentatively recognized six species, although unpublished work (C. F. Kammerer, 2023, in preparation) indicates that few of these are valid, and recognizes only a single species of *Gorgonops* (the type, *G*. *torvus*) as occurring in the *Endothiodon* Assemblage Zone of the Karoo Basin. Sigogneau-Russell (1989, p. 88) diagnosed *G*. *torvus* on the basis of “Middle size. Wide supraorbital frontal. Pterygoid transverse apophyses with teeth and rather posteriorly situated. Mandibular symphysis somewhat sloping. Five postcanines.” This diagnosis is accurate, but insufficient to identify *Gorgonops* even at the generic level. While recognizing that specific autapomorphies for *G*. *torvus* must wait for revision of the *Gorgonops* complex, we consider the genus to be diagnosable based on the combination of 4–5 upper postcanines, a ‘step’-like feature anterior to the canine on the ventral margin of the maxilla, a lobate interchoanal body of the vomer, delta-shaped palatine bosses, dentition on the palatine bosses, palatal bosses of the pterygoid, and pterygoid transverse processes, ‘backswept’ transverse processes, and a postorbital bar anteroposteriorly expanded and rugose, but not pachyostosed. *Gorgonops* specimens generally exhibit a relatively long, low snout, but these proportions are commonly altered by taphonomic crushing (as appears to be the case for SAM-PK-K10591). With the caveat that the very limited postcranial record of gorgonopsians complicates recognition of skeletal apomorphies, potentially diagnostic postcranial characters for this species include: triangular radiale and ultimate phalanges shorter than ulnare in the manus, an anterior groove on medial surface of ilium, a large posterior depression on medial surface of ischium, and a weakly pronounced ventral distinction between pubis and ischium.

**Holotype**

NMHUK PV R 1647. Incomplete and dorsoventrally flattened skull, see Owen (1876b).

**List of comparative materials**

AMNH FARB 2240, *Lycaenops ornatus*; BSPG 1934 VIII 28, 522–540, *Gorgonops* sp. (“*Scymnognathus* cf. *whaitsi*”); BP/1/2167, *Dinogorgon rubidgei*; BP/1/1210, unidentified gorgonopsian (likely rubidgeine; historically labeled *Dinogorgon*?); BP/1/4089, *Gorgonops torvus*; CGS FL 14, *Lycaenops ornatus*; GPIT-PV-31579, “*Scymnognathus*” *parringtoni*; NHCC LB350, *Aelurognathus tigriceps*; NHCC LB1073, unidentified gorgonopsian; NMHUK PV R 1647, *Gorgonops torvus*; PIN 156/5, *Sauroctonus progressus*; PIN 2005/1758, *Inostrancevia alexandri*; PIN 2212/61, *Viatkogorgon ivakhnenkoi*; PIN 2351/32, *Inostrancevia latifrons*; PIN 4548/1, *Suchogorgon golubevi*; RC 60, *Aelurognathus tigriceps*; SAM-PK-2342, *Aelurognathus tigriceps*; SAM-PK-9344, “*Aelurognathus*” *microdon*; SAM-PK-9345, *Arctognathus curvimola*; SAM-PK-K10539, *Cyonosaurus* sp.; UMZC T883, unidentified gorgonopsian; USNM 412381, *Cyonosaurus* sp.

## Materials and Methods

**Figure 1:**
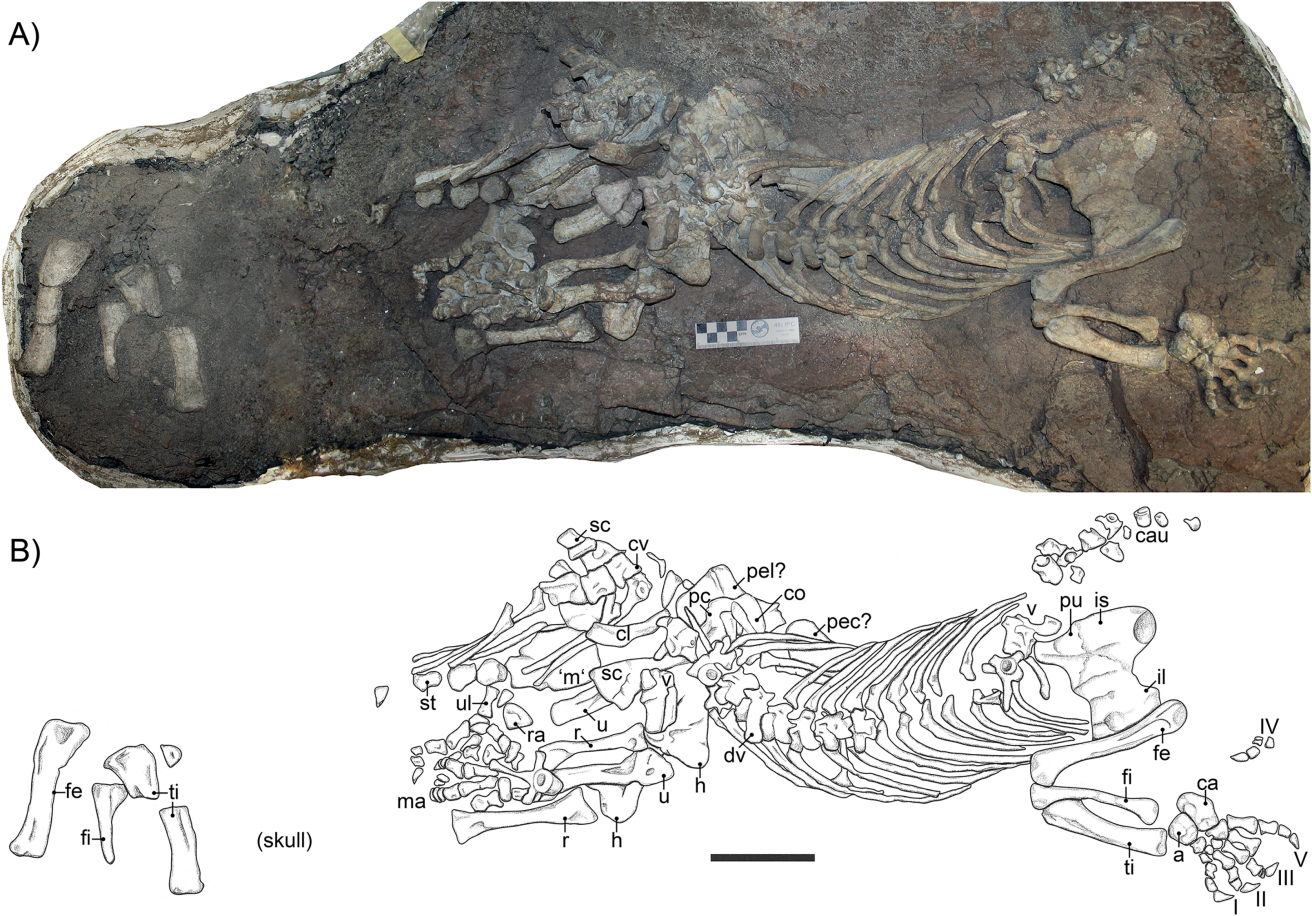
(A) photograph and (B) interpretative drawing of the skeleton of specimen SAM-PK-K10591 (*Gorgonops torvus*). Scale bars = 10 cm. Abbreviations: a, astragalus; ca, calcaneum; cau, caudal vertebrae; cl, clavicle; co, coracoid; cv, cervical vertebra; dv, dorsal vertebra; fe, femur; fi, fibula; h, humerus; is, ischium; ‘m’, manubrium; ma, manus; pc, procoracoid; pec?, pectoral girdle; pel?, pelvic girdle; pu, pubis; r, radius; ra, radiale; sc, scapula; st, sternebrae; ti, tibia; u, ulna; ul, ulnare; v, vertebra. Roman numerals indicate positions of pedal digits.

The specimen was found *in situ* by Georgina Farrell in 2007 and excavated in a single block (Fig. 1). The skeleton is almost complete. Two skulls were associated with SAM-PK-K10591, but the alternate skull, found *ex situ*, was discarded as a possible match for the skeleton of SAM-PK-K10591, mainly due to its small size. Mechanical preparation was performed initially by Georgina Farrell and later by Nyaniso Nofingxana, and the specimen is housed in the collections of the Iziko South African Museum, Cape Town.

**Locality.** SAM-PK-K10591 was found on the farm Wilgerbosch Kloof 449, south of Fraserburg in the Northern Cape Province, South Africa (also see Bendel et al. (2022)). Fossil-bearing exposures at this locality pertain to the lower part of the *Tropidostoma-Gorgonops* Subzone of the *Endothiodon* Assemblage Zone (AZ) (Botha & Angielczyk, 2007; Day & Smith, 2020; Kammerer & Smith, 2017).

*Gorgonops torvus* has also been reported from the higher stratum of the *Cistecephalus* AZ (Sigogneau-Russell, 1989), but these and earlier records (from the *Tapinocephalus* AZ) require re-examination in the context of a comprehensive revision of the genus *Gorgonops*, due to historically uncertain stratigraphy of this portion of the Beaufort Group (Day & Rubidge, 2020). A more detailed account of the taphonomy of SAM-PK-K10591 can be found in Bendel et al. (2022).

**Institutional abbreviations**. AMNH FARB, American Museum of Natural History, Fossil Amphibian, Reptile, and Bird Collection, New York, USA; BP, Evolutionary Studies Institute (ESI; formerly Bernard Price Institute for Palaeontological Research), University of the Witwatersrand, Johannesburg, South Africa; BSPG, Bayerische Staatssammlung für Paläontologie und Geologie, Munich, Germany; CGS, Council for Geoscience, Pretoria, South Africa; GPIT/IGP, Paläontologische Sammlung, Eberhard Karls Universität Tübingen, Tübingen, Germany; NHCC, National Heritage Conservation Commission, Lusaka, Zambia; NMHUK, Natural History Museum, London, UK; PIN, Paleontological Institute of the Russian Academy of Sciences, Moscow, Russia; RC, Rubidge Collection, Wellwood, Graaff-Reinet, South Africa; SAM, Iziko South African Museum, Cape Town, South Africa; UMZC, University Museum of Zoology, Cambridge, UK; USNM, Smithsonian Institution, National Museum of Natural History, Washington, USA.

## Description

The skeleton is fairly well preserved in general, though some elements are missing and others are damaged (*e.g*., the scapulae and interclavicle). The preservation of the skull is wanting. The entire postcranial skeleton stretches out over a block measuring 230 cm × 65 cm × 25 cm. Some pre-burial disturbance of the skeleton occurred, which has been attributed to scavenging (see also Bendel et al., 2022).

### Skull and mandible

**Figure 2:**
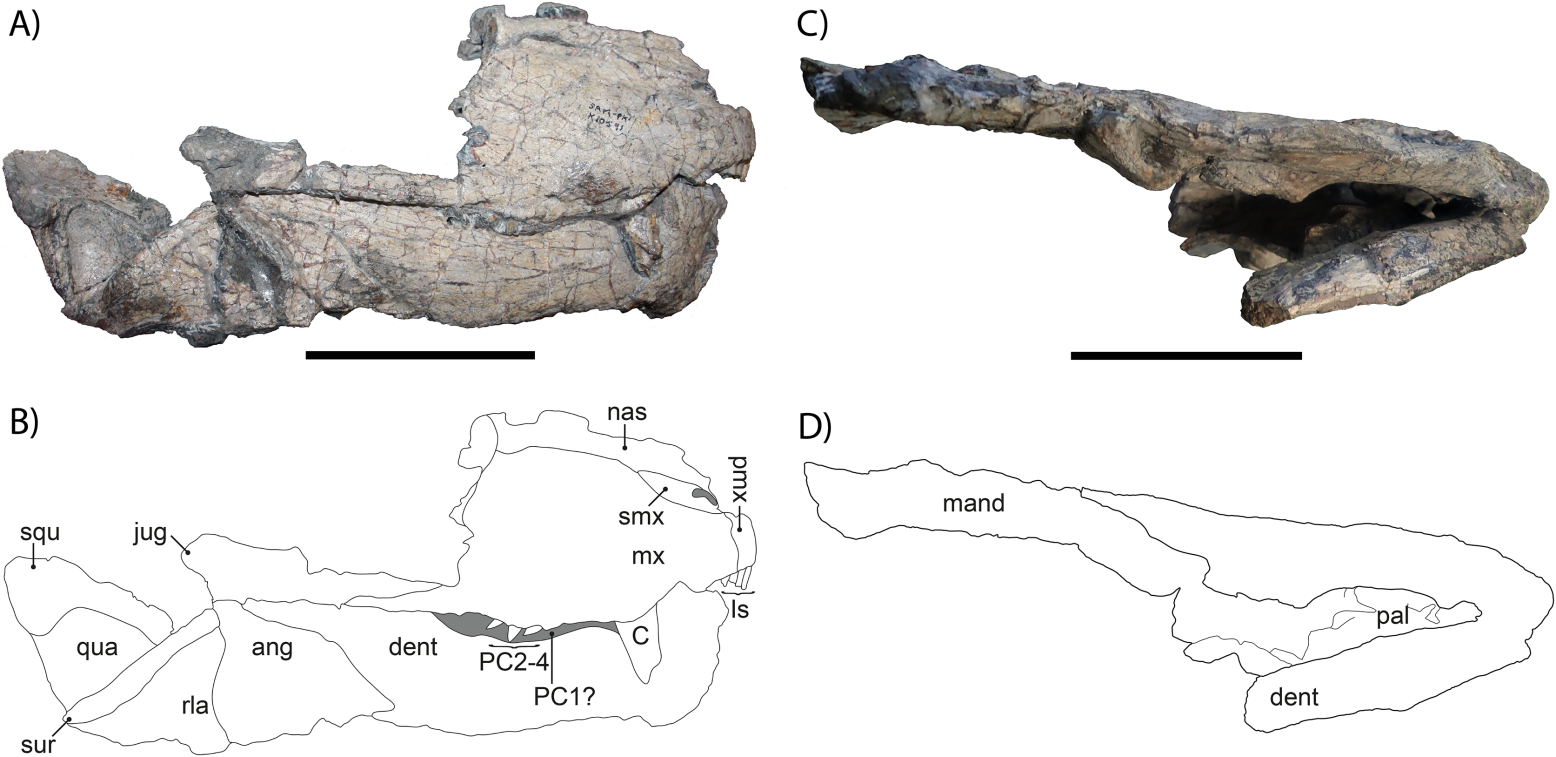
(A) Photograph and (B) drawing of the right lateral side of the skull of *Gorgonops torvus*, (C) photograph and (D) drawing of the ventral side of the skull of *Gorgonops torvus*. Scale bars = 10 cm. Abbreviations: ang, angular; C, canine; dent, dentary; Is, incisors; jug, jugal; mand, mandibular post-dentary bones; mx, maxilla; nas, nasal; pal, palate; PC1-4, postcanines; pmx, premaxilla, qua, quadrate; rla, reflected lamina; squ, squamosal; sur, surangular and articular.

The skull and mandible of SAM-PK-K10591 (Fig. 2) are incomplete and show signs of lateral crushing. The skull was discovered in association with the postcrania, between the right hindlimb and the rest of the skeleton (see Fig. 1), but has since been prepared off the block. The overall dimensions of the skull of SAM-PK-K10591 are slightly higher than wide, but this is due to lateral crushing of the cranium, which is indicated by disarticulation of the mandibular elements. Typically, the skull of *Gorgonops torvus* is wider than tall (Sigogneau-Russell, 1989), and variance from this in SAM-PK-K10591, which is preserved in a lateral-up attitude, is likely attributable to post-burial compaction (Bendel et al., 2022).

The anterior portion of the snout is preserved, but posteriorly broken off on the right side, missing most of the prefrontal, lacrimal, and facial portion of the jugal. On the left side, the skull is broken through the maxilla. Overall, the right side of the snout is in much better condition than the left. The same is true for the mandible, which is almost complete on its right side but broken off in the middle of the dentary on the left side. Due to poor preservation and obscuring by the occluded mandible, there is unfortunately little information on this specimen’s palate.

The skull is not pachyostosed and when intact would have measured roughly 30 cm in length (exact measure not possible due to damage), suggesting an adult gorgonopsian by comparison with other specimens of *Gorgonops*. The snout is relatively long (~13 cm until break in preorbital region) and the premaxilla bears the typical five incisors present in most gorgonopsians. The canine is by far the largest tooth in the snout, but its total length is uncertain due to breakage. The upper postcanine count is probably four, based on the better-preserved right maxilla, complying with the number reported for *Gorgonops torvus* by Kammerer et al. (2015), and in contrast to Sigogneau-Russell (1989), who listed five postcanines as diagnostic for this species. Both counts are likely accurate for *G*. *torvus*, reflecting minor individual variation of the sort common in early theriodonts.

The lateral borders of the skull are smooth and broaden slightly posteriorly; no deep depression or deflection is present on the facial portion of the jugal or the zygomatic arch. As is the case for many gorgonopsians, the skull widens posteriorly (Sigogneau-Russell, 1989). The septomaxilla is directed posterodorsally. The ventral border of the maxilla exhibits a small, step-like, ventrally directed bend anterior to the canine, which is pronounced on both sides of the skull, as seen in other specimens of *G*. *torvus* (*e.g*., BP/1/4089). The posterior process of the maxilla, which projects under the ventral border of the jugal, is short, although no more detailed information can be given due to breakage.

The lacrimal and prefrontal are not preserved at all, and the subtemporal extension of the jugal is not fully preserved, so no definitive information can be given about its length or height. The quadrate is blocky and roughly triangular, and the squamosal is only partially preserved. The squamosal sulcus extends onto the lateral surface of the skull, as in most gorgonopsians (Kammerer, 2016). The surangular is only visible as a slender ridge on the posterodorsal jaw margin.

The chin is of medium height and sharply sloped (ca. 60°). Under the freestanding coronoid process is the reflected lamina of the angular, which bears a strongly pronounced vertical and a less distinct horizontal ridge, which is characteristic for Gorgonopsia (Olroyd & Sidor, 2022).

The combination of characters observed in the cranium of this specimen (particularly the combination of four postcanines with a relatively long snout bearing a precanine ‘step’) is most consistent with *Gorgonops torvus* among *Endothiodon* AZ gorgonopsians, and it is referred to that species here. However, additional cranial-postcranial associations are needed to confirm that the cranial characters diagnostic for *Gorgonops* have a 1:1 association with the distinctive postcranial characters of SAM-PK-K10591.

### Vertebrae

The number of gorgonopsian presacral vertebrae has been described as 27+/−1, similar to therocephalians (Attridge, 1956). They have generally been considered to represent seven cervical and 20 dorsal vertebrae (Sigogneau-Russell, 1989), with minor variations of seven and 19 in *“Scymnognathus*” *parringtoni* (Gebauer, 2014) and seven and 21 in *Viatkogorgon ivakhnenkoi* (Tatarinov, 2004). There is no clear distinction between the last cervical and first dorsal vertebrae, and the recognition of seven cervicals (see *e.g*., Müller et al., 2010) is based largely on inferred homology with cynodonts. Following the dorsal series, three sacral vertebrae connect to the ilium. The posteriormost vertebrae consist of a variable numbers of caudals (15 preserved in *Inostrancevia* (Sigogneau-Russell, 1989), 16 in an unidentified Tanzanian gorgonopsian (UMZC T883, E. Bendel, C. Kammerer, 2017, personal observations), and 20 in *Viatkogorgon* (Tatarinov, 2004)).

**Figure 3:**
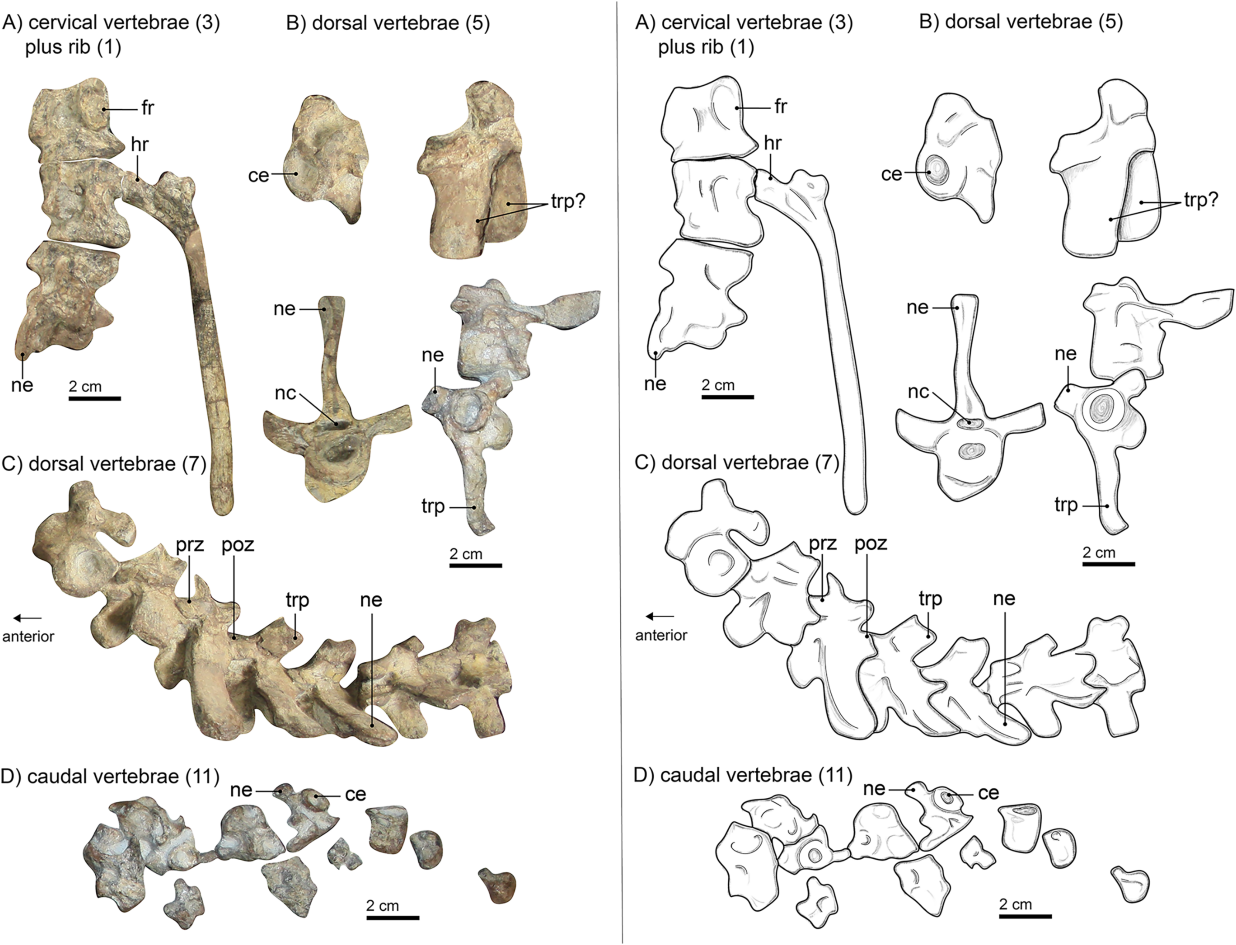
Left panel: (A) photograph of cervical vertebrae, (B) of isolated dorsal vertebrae, (C) of articulated dorsal vertebrae and (D) of caudal vertebrae of *Gorgonops torvus*. Right panel: interpretative drawings of same Scale bars = 2 cm. Abbreviations: ce, centrum; fr, facet for head of rib; hr, head of rib; nc, neural canal; ne, neural spine; poz, postzygapophysis; prz, prezygapophysis; trp, transverse process.

In SAM-PK-K10591, only six vertebrae (E. Bendel, C. Kammerer, 2017, the anterior dorsal series) are still preserved in articulation. A maximum number of 26 vertebrae can be identified, of which three are likely cervicals, 12 are dorsals, and 11 are caudals. The latter are mostly distinguished by size and their location on the fossil slab (Figs. 1 and 3). All identifiable vertebrae are amphicoelous. No intercentra are visible, although they are known in other gorgonopsians (*e.g*., in *Lycaenops ornatus* (Colbert, 1948) or *Inostrancevia* (Pravoslavlev, 1927a)). However, as the only articulated vertebrae are preserved dorsal-up, it is possible that they are present but still embedded in the matrix.

Neither the atlas nor the axis is preserved. This is in concordance with the skull being only partially preserved and not found in direct articulation with the rest of the skeleton (see Materials and Methods; also Bendel et al. (2022)). Three articulated cervical vertebrae (Fig. 3A) are preserved near the sternum. They appear to show consistent morphological differences from the articulated vertebrae lying between the specimen’s ribs. The former are stouter and the transverse processes are considerably shorter and more rounded in cross-section than those of the latter. Only a partial neural spine is preserved on the most posterior of the three articulated elements. It is directed slightly posteriorly. The pleurocentra are massive. The posterior-most centrum is the anteroposteriorly longest of the three and all are round in cross section.

The dorsal vertebrae (Figs. 3A–3C) are spread over the manus, the humerus, and the ribs. Six of them are articulated, two more are disarticulated in positions anterior to the others, and two more are located near the pelvis. Their locations and size suggest that they are part of the anterior dorsal column. All dorsals have strongly amphicoelous centra, which are smaller than their cervical counterparts. For comparison, the best-preserved cervical vertebra (the second) has a centrum with a diameter of roughly 3 cm and is 3.2 cm in maximum anteroposterior length. The best preserved and largest dorsal vertebra has a centrum of ca. 1.8 cm diameter and is about 2.4 cm long. The neural spines are long (up to 3.5 cm) and slender but become slightly shorter and more posteriorly inclined towards the pelvis. This is typical for gorgonopsians (Sigogneau-Russell, 1989). Although the neural spines in the holotype of *Lycaenops ornatus* (AMNH FARB 2240, Colbert, 1948) are consistently short, these were reconstructed in plaster and likely do not reflect this specimen’s original morphology, as demonstrated by other *L*. *ornatus* specimens (*e.g*., CGS FL 17) preserving intact, longer neural spines. The proportions of vertical centrum diameter and length of the neural spine are like those of *Inostrancevia alexandri* (Pravoslavlev, 1927b). The transverse processes are flatter than the neural spines and more massive posteriorly as well (see articulated vertebrae *vs*. the disarticulated ones anteriorly) and are oval in cross-section. These proportions can also be observed in “*Scymnognathus* cf. *whaitsi*” (BSPG 1934 VIII 528). The pre- and postzygapophyses are consistent in size anteroposteriorly. The prezygapophyses are tilted dorsally at an angle of about 25° from the horizontal plane. The sacrum, typically consisting of three (sometimes fused) vertebrae, is not preserved in SAM-PK-K10591. The disarticulated preserved caudal vertebrae (Fig. 3D) are located above the pelvic girdle and are smaller than the anterior vertebrae, decreasing more in total size posteriorly, with progressively reducing centrum diameter and neural spine length (if the latter is present at all). The total length of the tail is unknown.

### Ribs

**Figure 4:**
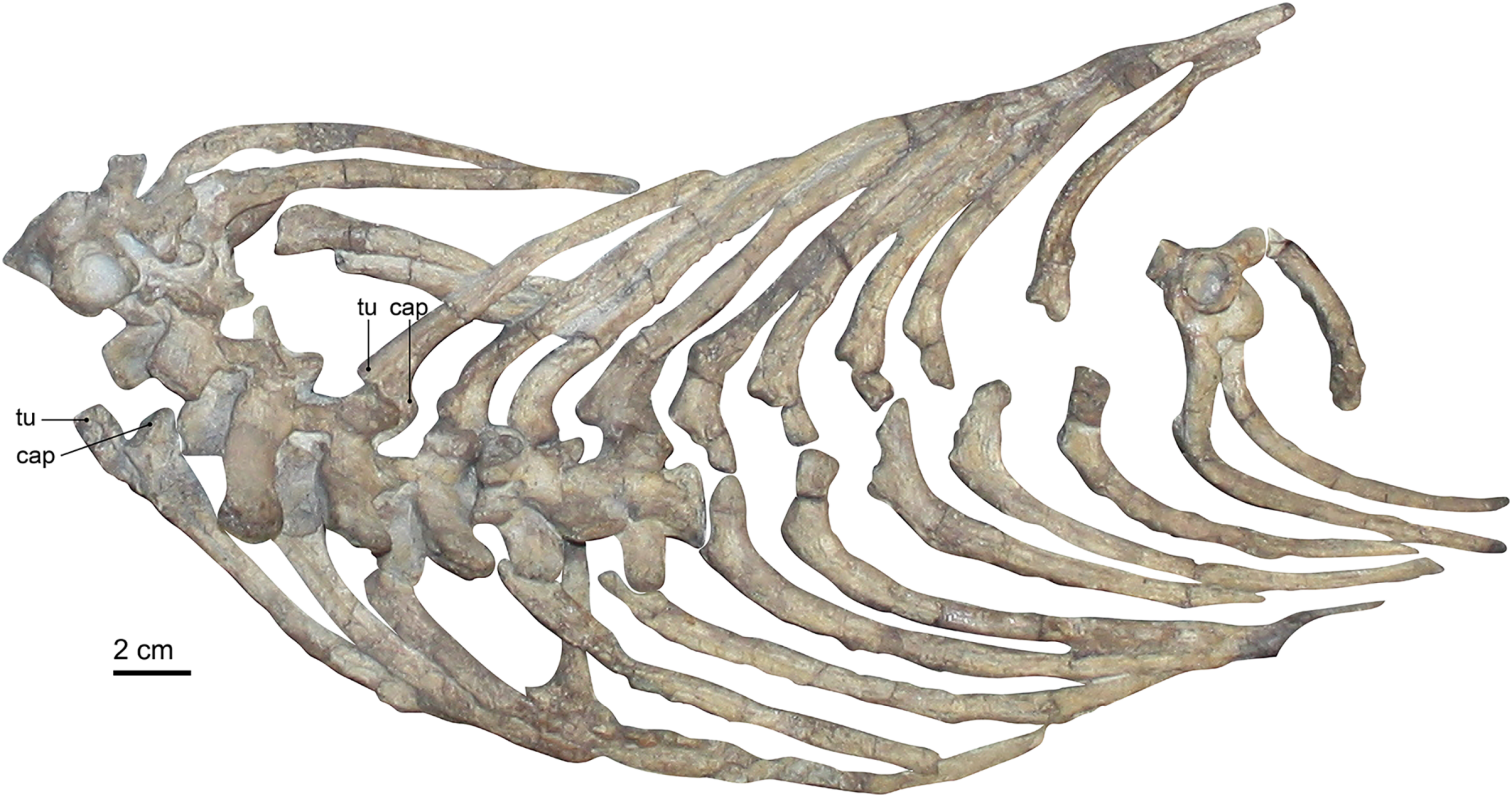
Photograph of the rib cage of *Gorgonops torvus*. Scale bar = 2 cm. Abbreviations: cap, capitulum; tu, tuberculum.

The preserved ribs of SAM-PK-K10591 are long and slender (Figs. 1 and 4). They are mostly still in articulation, or, in the case of the posterior dorsals, still in their original positions although the associated vertebrae have been displaced. Some additional ribs are loosely scattered across the rest of the skeleton, and a few are only preserved as fragments. The longest preserved ribs are 15–17 cm long. In general, no substantive difference in length of the ribs can be noted, but the posteriormost dorsal rib, which is disarticulated, appears somewhat shorter and thicker than the others. It is only partially exposed, and its differing morphology could indicate that it is a lumbar rib, although it may also just be broken off distally.

All ribs are very clearly bifurcated to contact the vertebrae at their transverse processes and parapophyseal facets. This bicipital morphology is less pronounced in the posterior ribs towards the pelvis. The length and facets of the sternum suggest an articulation of the ribs with the sternum *via* costal cartilages, although no such articular contact has been preserved. Boonstra (1934b), Broom (1932), and Colbert (1948) could not confidently determine whether the ribs continued up to the sacrum in the gorgonopsian specimens they studied. In other gorgonopsians, such as *Viatkogorgon* (Tatarinov, 2004) and an unpublished *Cyonosaurus* specimen (SAM-PK-K10539, C. Kammerer, E. Bendel, 2018, personal observations), some posterior ribs are present. They are shorter than the previous ribs and appear to form a ‘lumbar’ series (albeit without the clear differentiation in morphology seen in eutheriodonts). The absence of the sacrum itself in this specimen could be the reason why none (or potentially only one) of the ribs associated with the posteriormost dorsal vertebrae are found.

Compared to the ribs of “*Scymnognathus*” *parringtoni* (Gebauer, 2007), the ribs in SAM-PK-K10591 appear slightly thicker, comparable to the ribs in *Viatkogorgon* (Tatarinov, 2004) or “*Scymnognathus* cf. *whaitsi*” (Broili & Schröder, 1935). The articulation facet for the costal cartilage of “*Scymnognathus*” *parringtoni* seems to be thicker than in SAM-PK-K10591, even though this could be a preservational issue of the latter specimen. In all gorgonopsian ribs, however, the costal groove is very distinct and runs along the entire length of the individual bones.

The aforementioned ribs of *Viatkogorgon* are less well preserved in detail and overall shape. However, it is the only gorgonopsian specimen in which gastralia are present. Gastralia are sometimes referred to as ‘abdominal ribs’, although this term should be avoided since the developmental morphology of these structures is not homologous in all taxa (Claessens, 2004).

### Pectoral girdle

**Figure 5:**
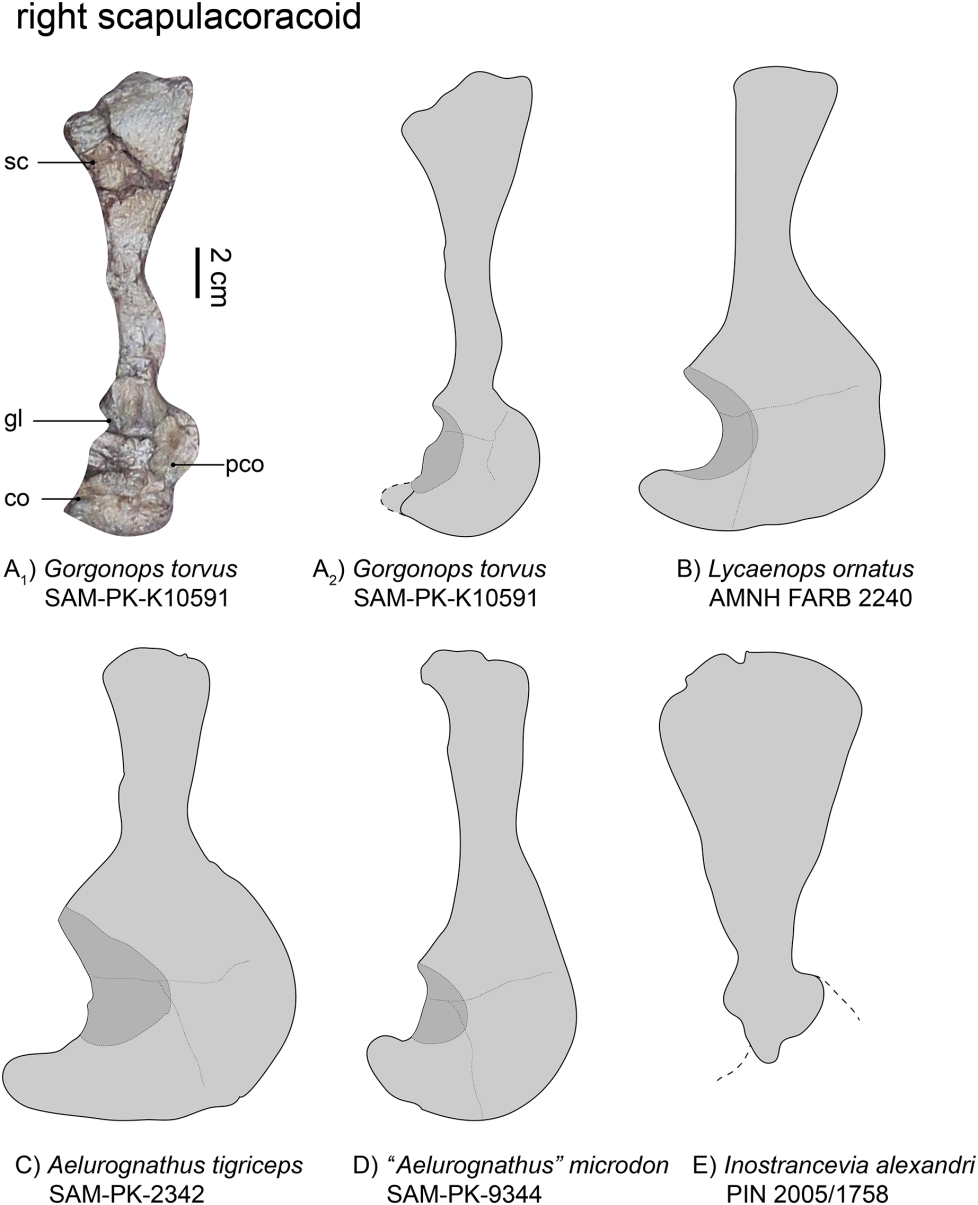
Comparison of various gorgonopsian scapulocoracoids. (A_1_) photograph of right scapulocoracoid of *Gorgonops torvus*, (A_2_) outline drawing of right scapulocoracoid *Gorgonops torvus*, (B) outline drawing of right scapulocoracoid of *Lycaenops ornatus* (mirrored left element), (C) outline drawing of right scapulocoracoid of *Aelurognathus tigriceps*, (D) outline drawing of right scapulocoracoid of *“Aelurognathus” microdon* (mirrored left element) and (E) outline drawing of right scapulocoracoid of *Inostrancevia alexandri* (mirrored left element). All in lateral view. Scale bars = 2 cm. Abbreviations: co, coracoid; gl, glenoid, pco, procoracoid, sc, scapula.

The typically fused elements of the scapula, procoracoid, and coracoid are mostly preserved in SAM-PK-K10591 (Figs. 1 and 5). No cleithrum can be identified. The right shoulder girdle consists of the scapular blade sticking out of the matrix (Fig. 1), with some of the procoracoid and coracoid discernible. The left scapula was mostly covered by the three articulated vertebrae at the top of the specimen, near the sternum. The removal of these vertebrae during further preparation (Bendel et al., 2022) was not able to reveal more of its morphology. An almost complete clavicle is situated near the anterior edge of the scapular blade. It is relatively short and thick (see Table S1), more like that of *Aelurognathus tigriceps* (Sigogneau-Russell, 1989) and *Lycaenops ornatus* (Colbert, 1948) than the longer, more gracile morphology observed in *Inostrancevia alexandri* (Pravoslavlev, 1927b), *Sauroctonus progressus* (Sigogneau-Russell, 1989), “*Scymnognathus*” *parringtoni* (Gebauer, 2014), or *“Aelurognathus*” *microdon* (Sigogneau-Russell, 1989).

The right scapula (Fig. 5), visible in lateral view, is broken and its dorsalmost portion was later reattached to the specimen. Due to this, the exact curvature of the blade is not preserved, but it was clearly concave medially. The dorsal-most border of the scapula is wide, almost double the width of the middle portion, although the narrowness of the latter has been exaggerated by taphonomic deformation (see Fig. 5A). Factoring this into consideration, the overall morphology of the scapula in this specimen appears most similar to that of *Lycaenops ornatus* (Colbert, 1948). In contrast, the scapular blade is extremely broad in *Inostrancevia alexandri* (Pravoslavlev, 1927b). In other Russian gorgonopsians, however, such as *Viatkogorgon ivakhnenkoi* (Tatarinov, 2004) and *Suchogorgon golubevi* (PIN 4548/1), the blade is much narrower. The dorsoventral height of the scapular blade is roughly comparable between SAM-PK-K10591 and “*Aelurognathus*” *microdon* (Boonstra, 1934b), although the latter has a narrower dorsal end. The scapula as a whole appears quite long; in terms of exposed elements it is almost triple the length of the procoracoid-coracoid complex, although the incomplete preservation of the latter likely underlies this ratio (see Table 1). The scapular blade is clearly proportionally longer than in some other African gorgonopsians, though, such as *Aelurognathus tigriceps* and *Arctognathus curvimola* (Sigogneau-Russell, 1989). Ventrally, the scapula exhibits a raised portion above the suture with the procoracoid. The contribution of the scapula to the glenoid appears only slightly smaller than that figured for *Lycaenops ornatus* (Colbert, 1948, and Fig. 3B). Both scapulae are very similar to that of “*Scymnognathus* cf. *whaitsi*” (BSPG 1934 VIII 540, Broili & Schröder, 1935).

**Table 1:** Ratios of maximum:minimum width of scapular blades in various gorgonopsian specimens.

	SAM-PK-K10591, *Gorgonops torvus*	SAM-PK-9344, “*Aelurognathus*” *microdon*	AMNH FARB 2240, *Lycaenops ornatus*	SAM-PK-2342, *Aelurognathus tigriceps*	GPIT-PV-31579, “*Scymnognathus*” *parringtoni*	PIN 2005/1758, *Inostrancevia alexandri*
Maximum:minimum width of scapular blade	1:2.5	1:1.7	1:1.3	1:1.5	1:1.8	1:3.8

The coracoid is typically an approximately triangular bone. It is mostly covered by two ribs such that its contribution to the glenoid fossa cannot be inspected in detail, but is predicted to be expansive based on the condition in other gorgonopsians (Sigogneau-Russell, 1989). The glenoid fossa, even though mostly obscured, appears neither as deep nor as wide as observed in other gorgonopsians (*e.g*., *Aelurognathus tigriceps*), rather similar to the shallow glenoid of SAM-PK-9344. The bone is flat and its most posterior process slightly rounded.

The triangular procoracoid appears small in SAM-PK-K10591, although it is potentially damaged at its anterior border. It is connected to both the scapula and the coracoid and its part in forming the glenoid fossa is probably minimal, as is typical for gorgonopsians (Sigogneau-Russell, 1989). The ribs blocking the glenoid fossa also cover a small depression which might be the lateral opening of the procoracoid foramen.

### Sternum and interclavicle

**Figure 6:**
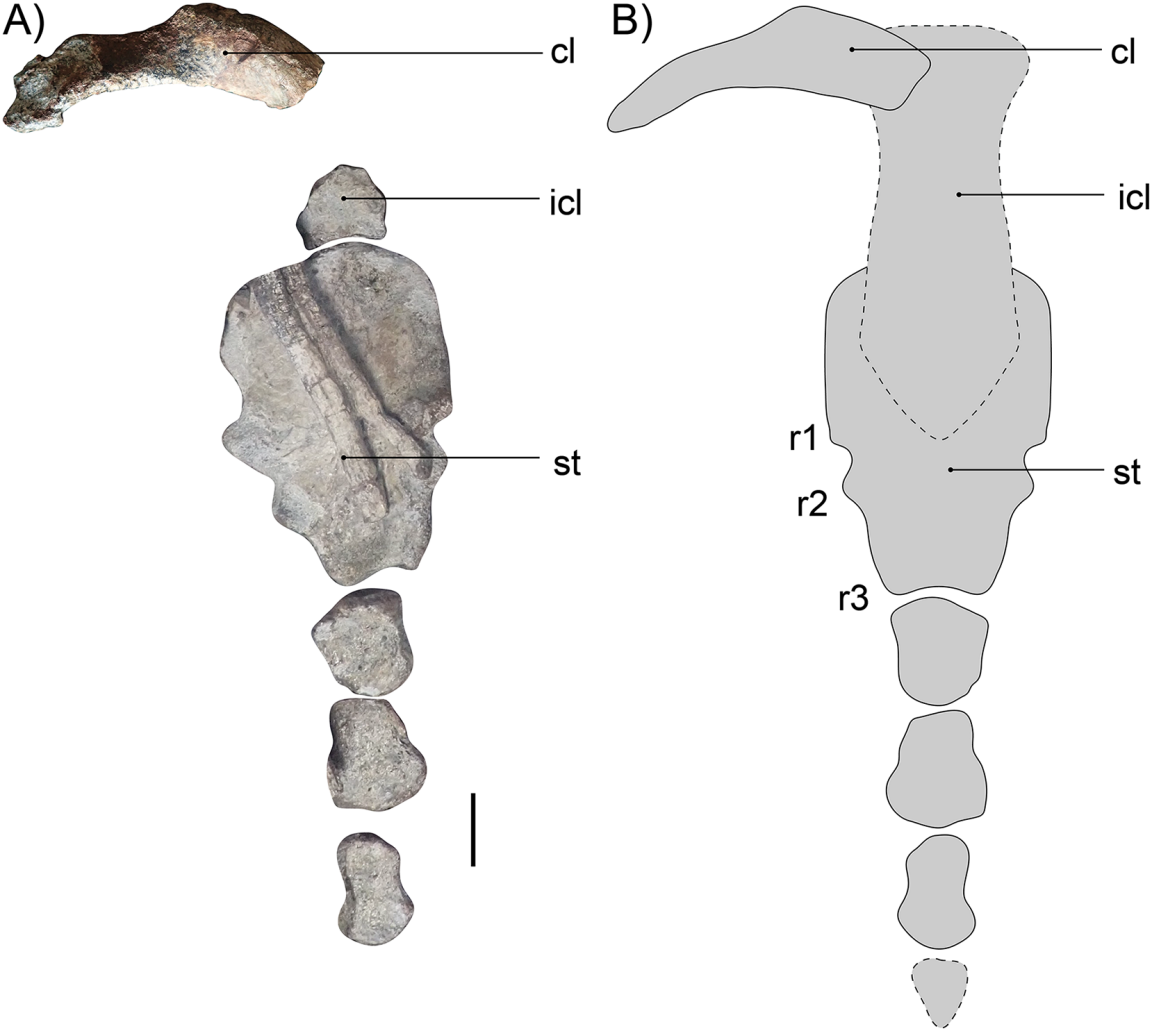
(A) Photograph and (B) reconstruction of sternum, interclavicle and clavicle of *Gorgonops torvus*. (A) from dorsal, with the clavicle moved from its original *in situ* position, (B) reconstruction from ventral with reconstruction of the ‘manubrium’ and cartilaginous rib articulation sites based on its right lateral side. Scale bar = 2 cm. Abbreviations: cl, clavicle; icl, interclavicle; r1-r3, rib articulation sites; st, sternum.

In the past, the sternal apparatus has been considered to constitute a single element throughout Gorgonopsia, variable in shape but usually a plate-like structure (Sigogneau-Russell, 1989). In contrast, SAM-PK-K10591 shows a much more complex apparatus (Bendel et al., 2022, and Fig. 6): the complete preserved sternum consists of four (but *in vivo* potentially five) elements, which are associated with a much less well-preserved anterior interclavicle. All parts are plate-like and are preserved dorsal-side up. The ‘manubrium’ is the largest element and roughly polygonal, similar in shape and size to the described sternum in *Aelurognathus tigriceps* (Broom, 1930; Sigogneau-Russell, 1989) and especially to the sterna of two specimens referable to *Cyonosaurus* (USNM 412381; see Bendel et al. (2022, Fig. S1B)) and *Aelurognathus* (NHCC LB350; C. Sidor, A. Mann, 2023, personal communications; Bendel et al. (2022, Fig. S1D)). The dorsal surface has an overall slightly concave curvature. Two trigonal protrusions for the potential articulation of costal cartilages are present on both lateral sides as well as in the contact area between the ‘manubrium’ and the first sternebra (r1–r3 in Fig. 6B). These protrusions are separated by a distinct notch in the lateral margin of the ‘manubrium’; the edge of this notch is raised, forming a dorsal rim connecting the two protrusions. Anterior to the more anterior protrusion, the dorsal surface of the sternum is depressed (visible in Fig. 6A). The left side of sternum appears to show a second lateral notch anterior to the protrusion. However, this may be artifactual (due to preparation and/or preservation) as the right side conforms with the reported morphology of *Aelurognathus* and *Cyonosaurus* (Sidor & Mann, personal communications) and is therefore more likely to reflect the true shape of the ‘manubrium’ (as reconstructed in Fig. 6B). Anterior to the protrusions, the ‘manubrium’ is transversely broad (widest near its anterior edge); posteriorly it narrows, with the posterior margin being roughly equal in width to the first two sternebrae.

At its posterior edge, the ‘manubrium’ exhibits a broad notch accommodating the first sternebra. In ventral view, the shape of the first sternebra is roughly pentagonal. The second sternebra is similar in shape and size to the first, whereas the third is slightly smaller and has a more pronounced lateral indentation on each side. The inter-sternebral points of contact (*via* cartilage) would also have been attachment sites for the ribs. As mentioned by Bendel et al. (2022), the anatomy of these axial elements is currently unique among known gorgonopsians, and is assumed to be the earliest occurrence of a multipartite sternum in synapsids. With this said, we consider it unlikely that this morphology is an autapomorphy of *Gorgonops torvus*. Given the paucity of gorgonopsian postcrania, particularly those including articulated pectoral girdles, and the high likelihood of disarticulation and loss for small, unfused elements like the sternebrae, we suspect that the absence of the latter in other specimens is mostly a preservational artifact. This is especially likely for other large gorgonopsians with a robust axial skeleton. For smaller gorgonopsians it is possible that sternebrae were cartilaginous and would not be found even in complete skeletons. It is worth mentioning that previous workers have predicted the presence of sternebrae in gorgonopsians: both Broom (1930) and Colbert (1948) inferred the potential presence of at least one additional element on the posterior border of the sternum due to the large articular surface in this area.

Only the posteriormost part of the interclavicle is preserved in SAM-PK-K10591 (Figs. 1 and 6). It is a small, platelike fragment. When intact, it would have had an articulation surface for the ‘manubrium’ dorsally and paired articulation surfaces for the clavicles ventrally (Bendel et al., 2022).

### Forelimb

**Figure 7:**
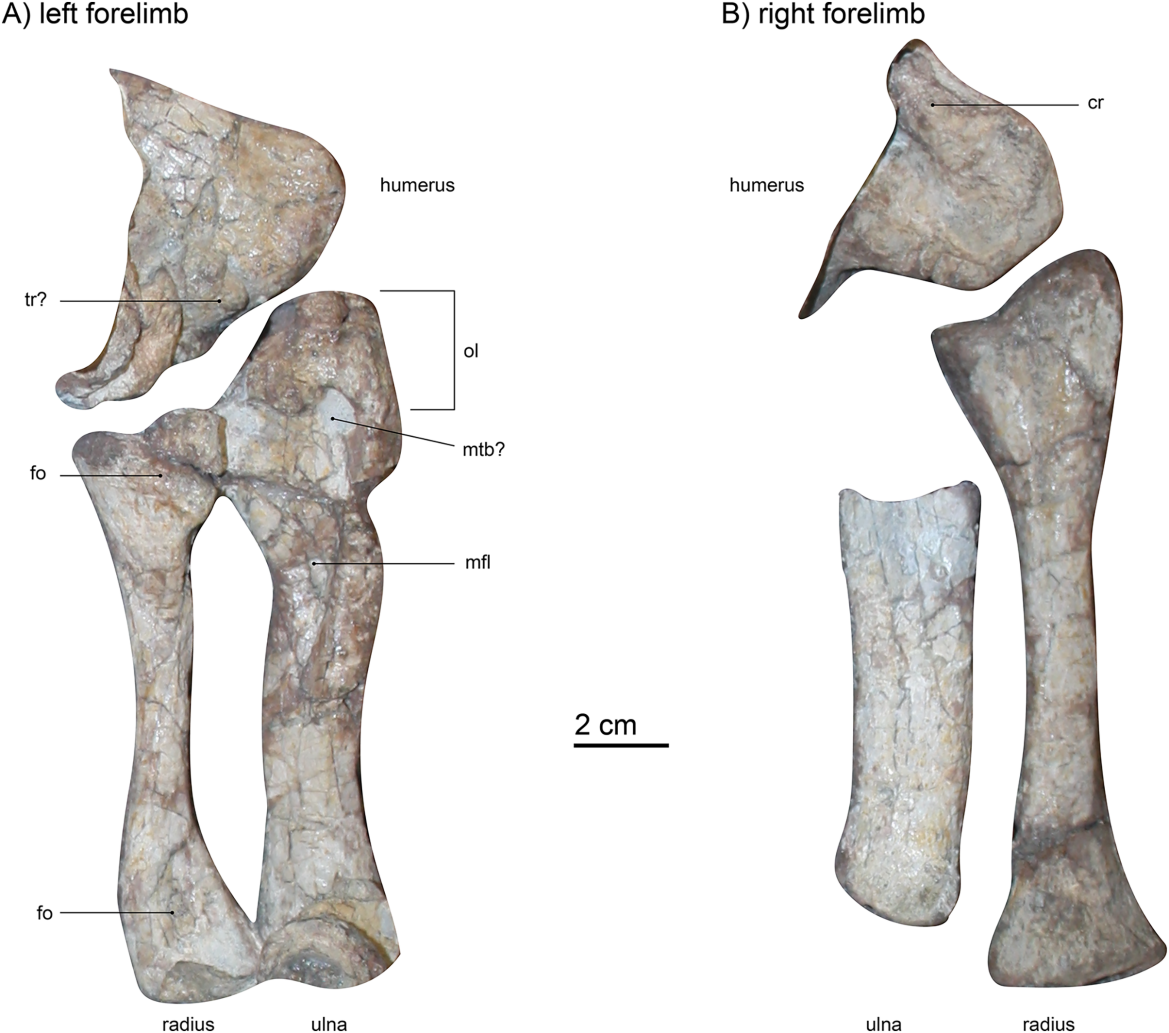
(A) left forelimb (dorsal view) and (B) right forelimb (ventral view) of *Gorgonops torvus*. Scale bar = 2 cm. Abbreviations: cr, crest; fo, fossa; mfl, attachment site for flexor muscles; mtb?, attachment site for musculus triceps brachii; ol, olecranon; tr?, trochlea.

**Humerus**.—Only the distalmost ends of both humeri are visible (Figs. 1 and 7). The left humerus (visible from dorsal) is hidden under some vertebrae and articulated with both radius and ulna through robust ent- and ectepicondyles. These rounded, distal condyles are typically massive in gorgonopsians (Sigogneau-Russell, 1989), as is the case for SAM-PK-K10591. However, they are less bulbous than in *Aelurognathus tigriceps* (SAM-PM-2342), more closely resembling the morphology of *Arctognathus curvimola* (SAM-PK-9345) or “*Scymnognathus* cf. *whaitsi*” (BSPG 1934 VIII 28). The right humerus (visible from ventral) is only partially preserved, lying under the left ulna, and is semi-articulated with the right radius. The entepicondylar foramen is covered by the ulna, but the lateral crest that would eventually extend to meet the dorsopectoral crest is exposed and quite massive, more so than in UMZC T883. This condition is similar to that in *Arctognathus curvimola* (SAM-PK-9345), even though the width of the distal humerus is not as extreme as in that specimen. Because of incompleteness, it is not possible to determine the degree of offset between the medial and distal ends of the humerus, which is generally 30–40° in gorgonopsians (Sigogneau-Russell, 1989). The distal condyles of the humerus are massive compared to the distal articulation areas in the femur, similar to that observed in *Lycaenops ornatus* (Colbert, 1948). The trochlea between the condyles is less visible in the right humerus and more pronounced in the left forelimb, acting as an articulation surface for the musculus triceps humeralis medialis (Angielczyk et al., 2009; Ray, 2006).

**Figure 8:**
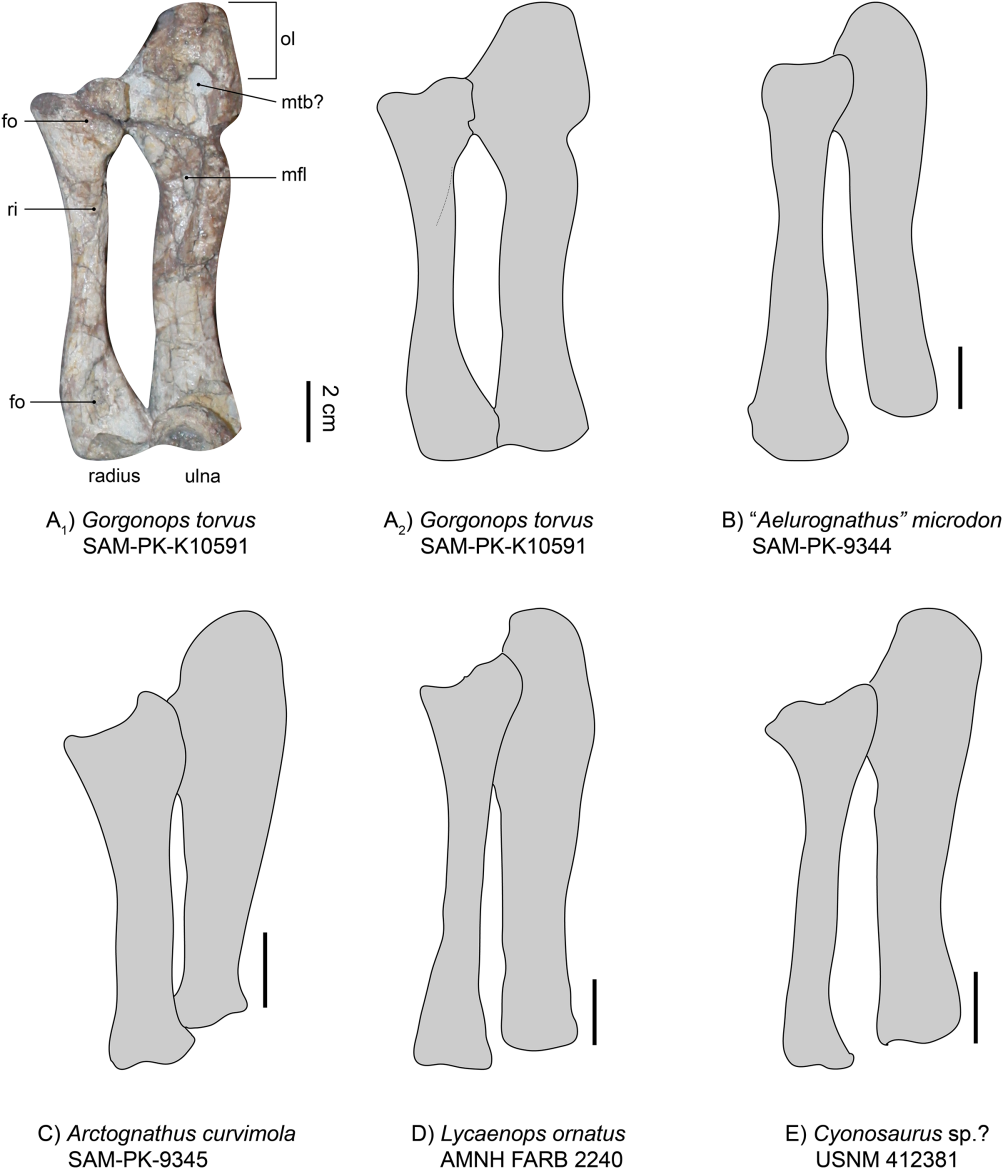
Comparison of various gorgonopsian radii and ulnae. (A_1_) photograph of left radius and ulna of *Gorgonops torvus*, (A_2_) outline drawing of left radius and ulna of *Gorgonops torvus*, (B) outline drawing of left radius and ulna of *“Aelurognathus” microdon*, (C) outline drawing of left radius and ulna of *Arctognathus curvimola* (mirrored right forelimb), (D) outline drawing of radius and ulna of *Lycaenops ornatus* and (E) outline drawing of radius and ulna of *Cyonosaurus* sp.? (mirrored right forelimb). All in medial view. Scale bars = 2 cm. Abbreviations: fo, fossa; mfl, attachment site for flexor muscles; mtb?, attachment site for musculus triceps brachii; ol, olecranon; ri, ridge for flexor attachment.

**Radio-ulnar-complex**.—The left ulna is a robust bone that is longer (proximodistal length 13.3 cm) than the associated radius (Figs. 7 and 8). Its dorsal side is facing upwards. The ulnar shaft has a roughly oval shape in cross-section. The right ulna is similar overall, though only the shaft is preserved. It is straighter and broader than the left ulnar shaft, probably due to crushing. On the left ulna, the proximal articulation surface with the humerus is broad in comparison to the shaft, and the olecranon is massive in width, but not very long. Roughly in the middle of the dorsal surface of the proximal ulna, an oval indentation is present, possibly forming a contact point for the musculus triceps brachii (Gambaryan & Kielan-Jaworowska, 1997). An elongate second indentation is located at the medio-dorsal edge for the articulation with the radius. From the middle of the shaft towards the proximal end, a very large depression can be found, directed medially. This fossa is sharply delimited and probably acted as a muscle attachment site for the flexor muscles of the ulna (Ray, 2006). In other gorgonopsians (apart from BSPG 1934 VIII 28) the margin of the fossa seems more diffuse and continues further proximally than in SAM-PK-K10591, *i.e*., in SAM-PK-9344 (“*Aelurognathus*” *microdon*) or AMNH FARB 2240 (*Lycaenops ornatus*). However, this may be an artefact of post-burial crushing. The distal condyle for the articulation with the ulnare is obscured by a vertebra overlying the left ulna and is not preserved in the right ulna.

The left radius (proximodistal length: 10.5 cm) is preserved in articulation with both the humerus and the ulna, is exposed in dorsal view, and is in good condition (Figs. 7 and 8). The medial proximal articulation surface is broken, but still preserved. The radius has a slim shaft, which is circular in cross-section as is typical for gorgonopsians (Sigogneau-Russell, 1989). The shaft appears straight in dorsal view, but curves and expands proximally and distally. This curvature appears to have been exaggerated by taphonomic deformation, accounting for the left radius being somewhat shorter than the right. Colbert (1948) reported the cross-section of the radius in *Lycaenops ornatus* as round, but it seems that a proximodistally oriented ridge projects from the middle of the proximal head to the medial side of the distal condyle on the dorsal surface of the bone. This ridge is only weakly developed in SAM-PK-K10591, and more so on its proximal end. In cynodonts, this ridge has been interpreted as the attachment site for a flexor muscle (Jenkins, 1971). Proximally, on the dorsal surface, a small fossa is present, just ventral to the slightly crescentic articular surface for the humerus. A wide, shallow indentation is preserved distally on the dorsal surface, near the contact with the radiale, possibly for muscular attachment. The right radius (proximodistal length: 13.3 cm) is visible from ventral and in addition to its absolutely greater length differs from the left radius in several proportional details. The right radius is straighter and more expanded at its proximal and distal ends, probably reflecting less deformation (it is more similar in shape to the radius in other gorgonopsians, particularly *Lycaenops ornatus*; Fig. 8). The ventral surface of the radius is smooth and almost featureless, apart from two portions of the bone flattening towards dorsal on the proximal as well as the distal ends, leaving a small crest between them on either side. On its distal end, it has a centrally raised area so that medially and laterally from it, small concave surfaces exist.

Overall, the relative dimensions (see Tables 2 and S1, and Fig. 8) of the radio-ulnar complex most resemble those described for *Lycaenops ornatus* and “*Scymnognathus*” *parringtoni*. *Arctognathus curvimola* is more stoutly built, whereas “*Aelurognathus*” *microdon* has a more slender ulna. However, post-burial vertical compaction of the bones could be a distorting factor. Minor variation in thickness aside, comparison of the general shape and proportions of the limb bones between the African taxa shows little variability. By contrast, clear distinction is present in the Russian *Inostrancevia alexandri* (Pravoslavlev, 1927b). It has a much stockier radius and ulna with a shorter total length compared to maximum width of the radial/ulnar head. The more slimly built anterior zeugopodium of the Russian *Viatkogorgon ivakhnenkoi* (Tatarinov, 2004) seems to follow the rather elongate and less robust bauplan of most Africa taxa.

**Table 2:** Different ratios of radius and ulna in various gorgonopsian specimens.

	SAM-PK-K10591, *Gorgonops torvus*	SAM-PK-9344, “*Aeluro-gnathus*” *microdon*	SAM-PK-9345, *Arcto-gnathus curvimola*	AMNH FARB 2240, *Lycaenops ornatus*	USNM 412381, *Cyono-saurus* sp.?	BSPG 1934 VIII 28	GPIT-PV-31579, “*Scymno-gnathus*” *parringtoni*	PIN 2005/1758, *Inostrancevia alexandri*
Radial head maximum width:ulnar head maximum width	1:1.5	1:1.2	1:1.3	1:1.1	1:1	N/A	1:1.4	1:1
Ulnar head maximum width:ulna length	1:3.2	1:3.6	1:2.9	1:3.9	1:3.5	1:4	1:3	1:1.4
Radial head maximum width:radius maximum length	1:4.2	1:3.9	1:3.5	1:4.1	1:3.4	N/A	1:3.9	1:2.8

**Figure 9:**
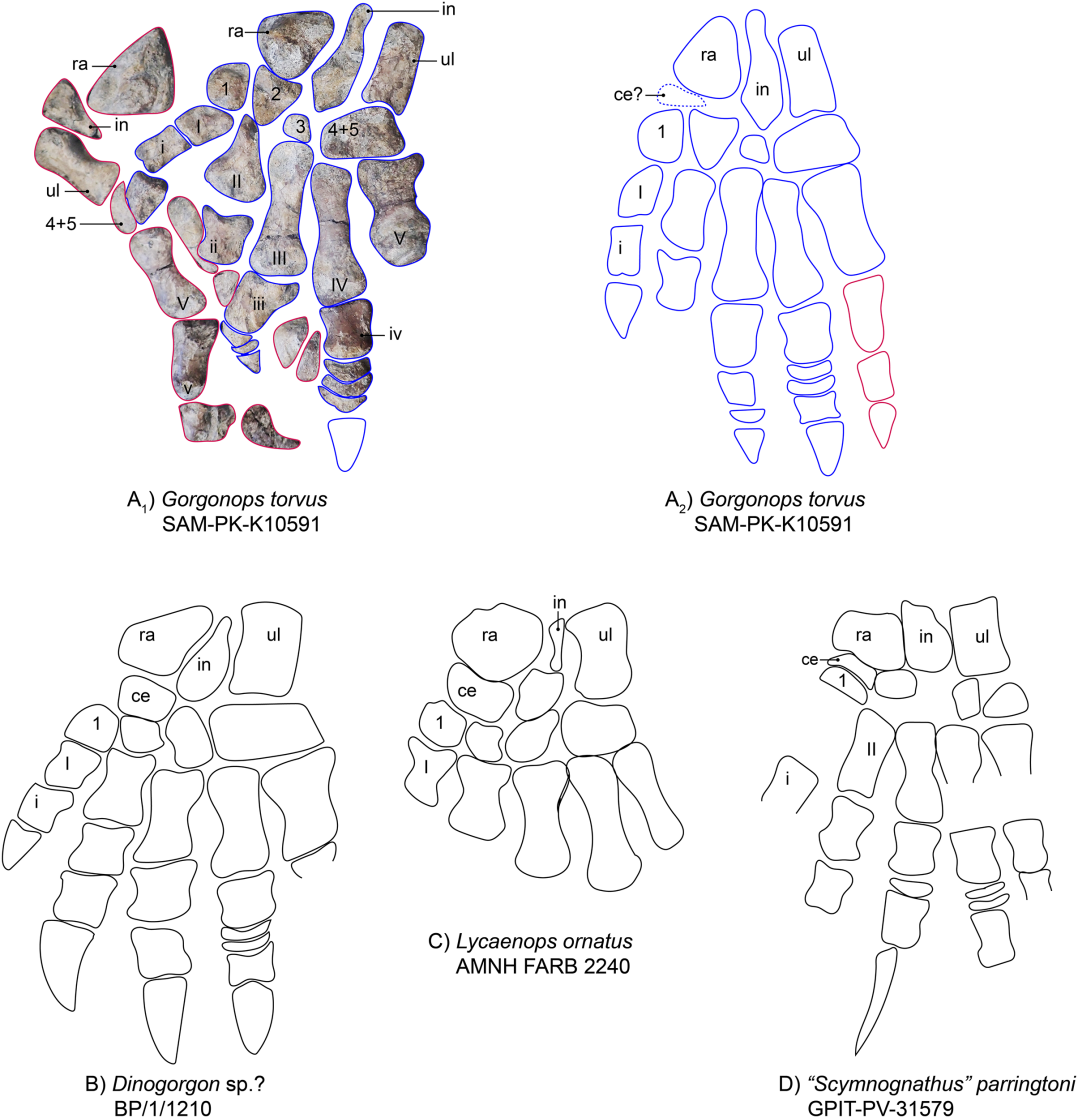
Comparison of various gorgonopsian hand skeletons. (A) Photograph of left (blue) and right (pink) manus of *Gorgonops torvus*, (B) idealized drawing of combined left hand skeleton of *Gorgonops torvus*, (C) idealized drawing of left manus of *Dinogorgon*sp.?, (D) idealized drawing of left manus of *Lycaenops ornatus* and (E) idealized drawing of left manus of “*Scymnognathus*” *parringtoni* (mirrored right manus). All in dorsal view. Abbreviations: ce, centrale; in, intermedium; ra, radiale; ul, ulnare.

**Manus**.—The left manus is almost completely preserved and visible in dorsal view (Figs. 1 and 9). One large vertebra obscured part of the left carpus, but even after preparing it off, the underlying parts remained unidentifiable. The carpus of the right hand is clearly visible, however. Both hands are quite robust and their morphology consistent with general gorgonopsian anatomy as previously described (*i.e*., Sigogneau-Russell, 1989, Fig. 9): The radiale is roughly triangular in outline and robust. This is an unusual feature, as the radiale is more rounded and rectangular in most other observed gorgonopsians (see Fig. 9 and Table S1). The radiale of *Aelurognathus tigriceps* (SAM-PK-2342) is also roughly triangular, but is more compressed proximo-distally. The ulnare is more elongate than the radiale and is slightly concave on both the medial and lateral edges, as in the other specimens investigated. In between the radiale and ulnare of the right hand, a medio-laterally constricted, wedge-shaped intermedium is present. The ulnare and intermedium both contact the merged 4^th^ and 5^th^ carpal bones distally, which seem to be fused in all gorgonopsian taxa (Hopson, 1995; Kümmell et al., 2020), *contra* (Sigogneau-Russell, 1989). This merged element is large and medio-laterally wider than the first three. No centrale is visible. Gorgonopsians usually have one or two centralia (Sigogneau-Russell, 1989), so their apparent absence may be preservational. As is typical for the group, the metacarpals are configured such that the fourth is the longest. The fifth is very stout. The other three decline in size from third to first. The fourth digit includes the most phalangeal elements: five including the displaced ungual, which could be the isolated claw-like element near the sternum. This is followed by the third digit, with four phalanges, which is only preserved in the left manus. The third and the fourth digit both possess disc phalangeal elements (one and two, respectively). The ungual phalanx appears shorter than that of *Arctognathus curvimola* (SAM-PK-3329) and especially “*Scymnognathus*” *parringtoni* (Sigogneau-Russell, 1989). All unguals are stouter than the ulnare in SAM-PK-K10591, whereas they are at least as long (BP/1/1210) or longer (GPIT-PV-31579) than the ulnare in the other two species in which they are preserved, at least in the third finger that all specimens preserve (see Fig. 9 and Table S1). For SAM-PK-K10591, the manual phalangeal formula is **2-?-4-5-3**.

The manual phalangeal formula is known for a wide range of gorgonopsian taxa and was long thought to differ between species (*i.e*., “*Scymnognathus*” *parringtoni* (Gebauer, 2014; Sigogneau-Russell, 1989)), *Aelurognathus tigriceps* (SAM PK-2342), *Lycaenops ornatus* (AMNH FARB 2240 (Colbert, 1948)), and an unidentified gorgonopsian (Rubidgeinae indet., BP/1/1210). Hopson (1995), however, reexamined most taxa and found the manual phalangeal formula to be quite consistent in gorgonopsians: 2-3-4-5-3. More recent studies of the synapsid manus have supported this count as standard for Gorgonopsia (*e.g*., Kümmell & Frey, 2014). The number of phalangeal elements in *Gorgonops torvus* thus comports with the usual state for the clade.

### Pelvic girdle

**Figure 10:**
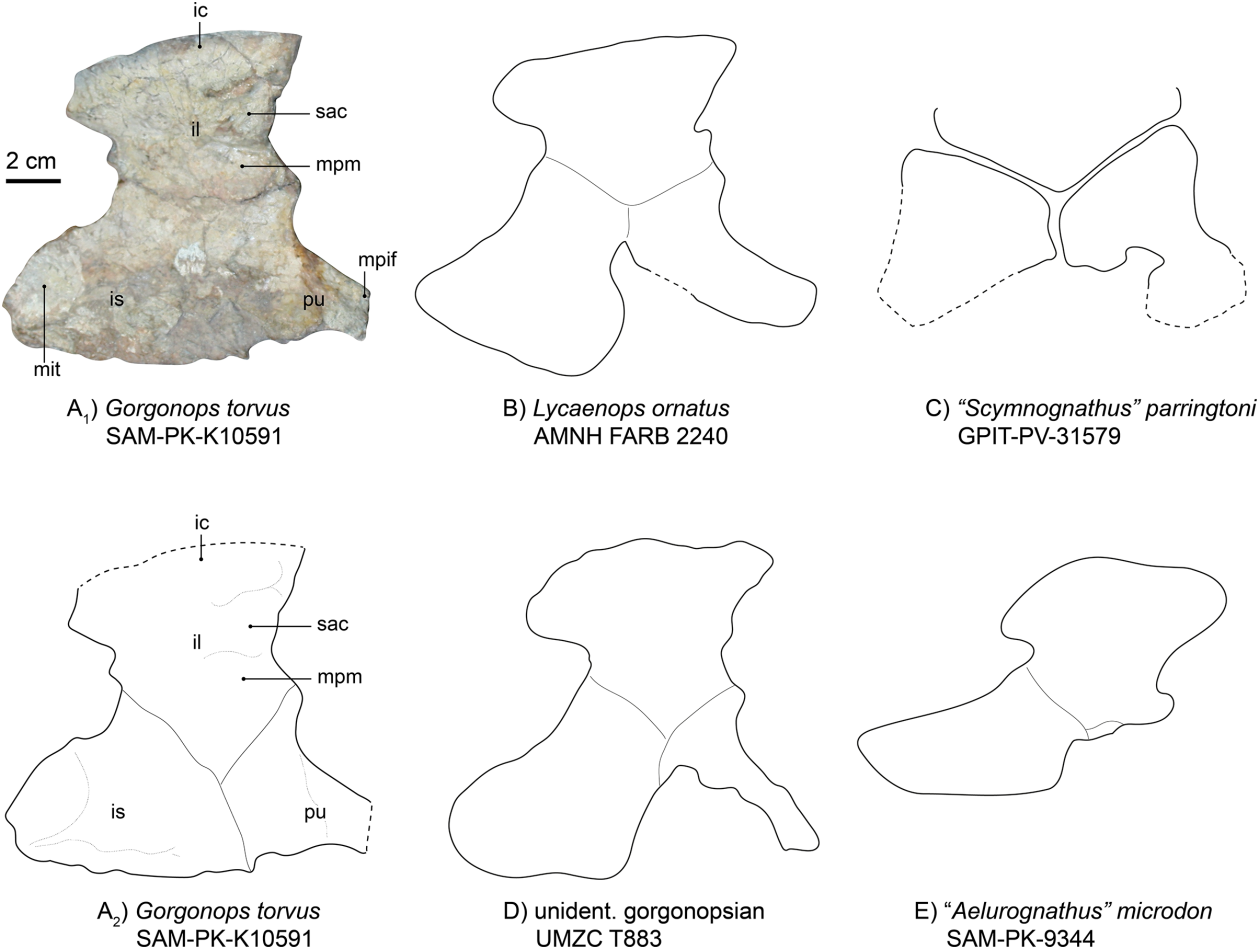
Comparison of various gorgonopsian pelves. (A_1_) Photograph of left pelvis of *Gorgonops torvus*, (A_2_) outline drawing of left pelvis of *Gorgonops torvus*, (B) outline drawing of left pelvis of *Lycaenops ornatus*, (C) outline drawing of left pelvis of *“Scymnognathus” parringtoni*, (D) outline drawing of pelvis of UMCZ T883 (mirrored right pelvis) and (E) outline drawing of left pelvis of *“Aelurognathus” microdon*. All in medial view. Scale bar = 2 cm. Abbreviations: ic, iliac crest; il, ilium; mit, attachment site for m. ischiotrochantericus; mpif, attachment site for m. puboischiofemoralis interior; mpm, attachment site for m. puboischiofemoralis, pu, pubis, sac, attachment for the first sacral vertebra.

The left innominate is reasonably well-preserved, visible in medial view and located near the associated left hind limb (Fig. 1). From what can be observed, this girdle element is almost completely flat. The iliac crest is broken off at its anterior tip and obscured by the femoral shaft, but is broad anteroposteriorly. Dorsoposteriorly, the ilium projects a bit beyond the body of the bone, creating a “neck” further towards the ischium where it is constricted again. The posterior angle of the constriction is about 115°, mirroring the condition in *Lycaenops ornatus* (Colbert, 1948) and UMZC T883, rather than in “*Aelurognathus*” *microdon* (SAM-PK-9344 (Sigogneau-Russell, 1989)) and “*Scymnognathus*” *parringtoni* (Gebauer, 2014), in which the angle is more acute, being close to 90° (see Fig. 10 and Table S1). This posterior process is also less tall and tapering compared to the aforementioned gorgonopsians. The medial surface of the plate-like bone features a complex relief, dominated by anteroposteriorly directed crests. One posterior crest causes the posterior process of the ilium to appear more massive than the anterior edge. Under the strongest pronounced crest on the anterior part of the bone, one less distinct facet for the articulation of the first sacral vertebra is present, under which, in turn, is the attachment area for the musculus psoas minor (Wright, 2018).

The ischium (Fig. 10) is the most massive part of the pelvic girdle and presents a large posterior extension. On this extension, a large oval depression is situated medially, making the ischium flatter in this area. Based on position, this depression may have acted as the attachment area for the musculus ischiotrochantericus. Ventrally, the depression is bordered by a ridge. Anterior to this depression, another anteroposteriorly elongated ridge continues until it reaches the pubis, where it flattens out. At its most ventral edge, the ischium becomes thinner. The overall shape of the ischium is different from that of *Lycaenops ornatus* in being more anteroposteriorly and less posteroventrally elongated. Furthermore, the entire ischial plate is smaller than in *Lycaenops*, but shows similarities in size to *Inostrancevia* (see Pravoslavlev, 1927b) and BSPG 1934 VIII 28. There is no obvious angle between its most anterior edge and the pubis; instead, they present a tight suture and are almost aligned with each other.

The pubis (Fig. 10) is missing the anterior section, probably due to its more elongated and thus more fragile nature. It has a broad suture with the ischium and a much narrower one with the ilium. It rapidly constricts towards anterior where the musculus puboischiofemoralis interior likely originated on its dorsal side (Wright, 2018). The anterior and ventral margins are concave. Anterior to the constriction, a dorsoventrally directed ridge is present.

Due to the attitude of preservation, the laterally situated acetabulum is not visible. Under the shoulder girdle, another plate-like element is preserved. Its large and blade-like structure strongly hints toward its association with the right pelvic girdle. Due to it being mostly concealed by the pectoral elements, however, no clear identification can be made.

### Hind limb

**Figure 11:**
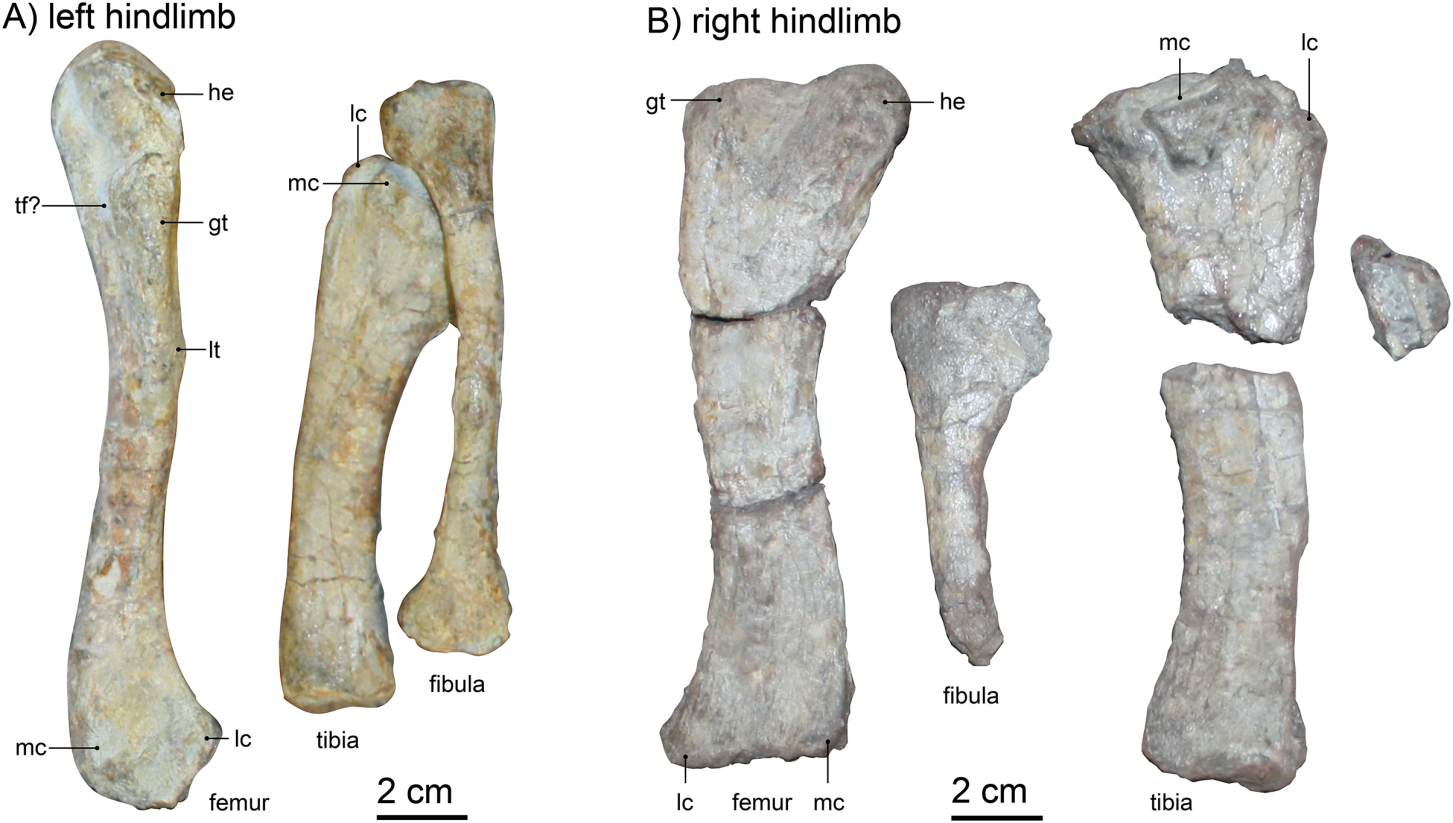
(A) left hindlimb and (B) right hindlimb of *Gorgonops torvus*. Femora in dorsolateral view, tibia and fibula in anterior view. Scale bar = 2 cm. Abbreviations: gt, greater trochanter; he: head; lc, lateral condyle; lt, lesser trochanter, mc, medial condyle; tf?, trochlear fossa.

**Femur**.—Both femora are preserved (Fig. 11), but only the left is located near its associated pelvis and articulated with the corresponding tibia-fibula complex (Fig. 1). Overall, the left femur is less crushed and in very good condition apart from some modern weathering, whereas the right is disarticulated and not preserved in such detail (Fig. 11). The left femur is lying with dorsal and lateral faces up, thus exposing the greater trochanter. The bone has a slight ‘S’-curvature and is quite slender, with a width of ~2 cm at the slimmest part of the diaphysis. The bone is weakly twisted, head offset at approximately 20°, hinting at a slight outward orientation of the femur from the hip. Ventrally, the left femur shows a mildly eroded lesser trochanter and a light crest for the attachment of femoral adductor muscles, reaching from the trochanter towards the distal metaphysis.

**Figure 12:**
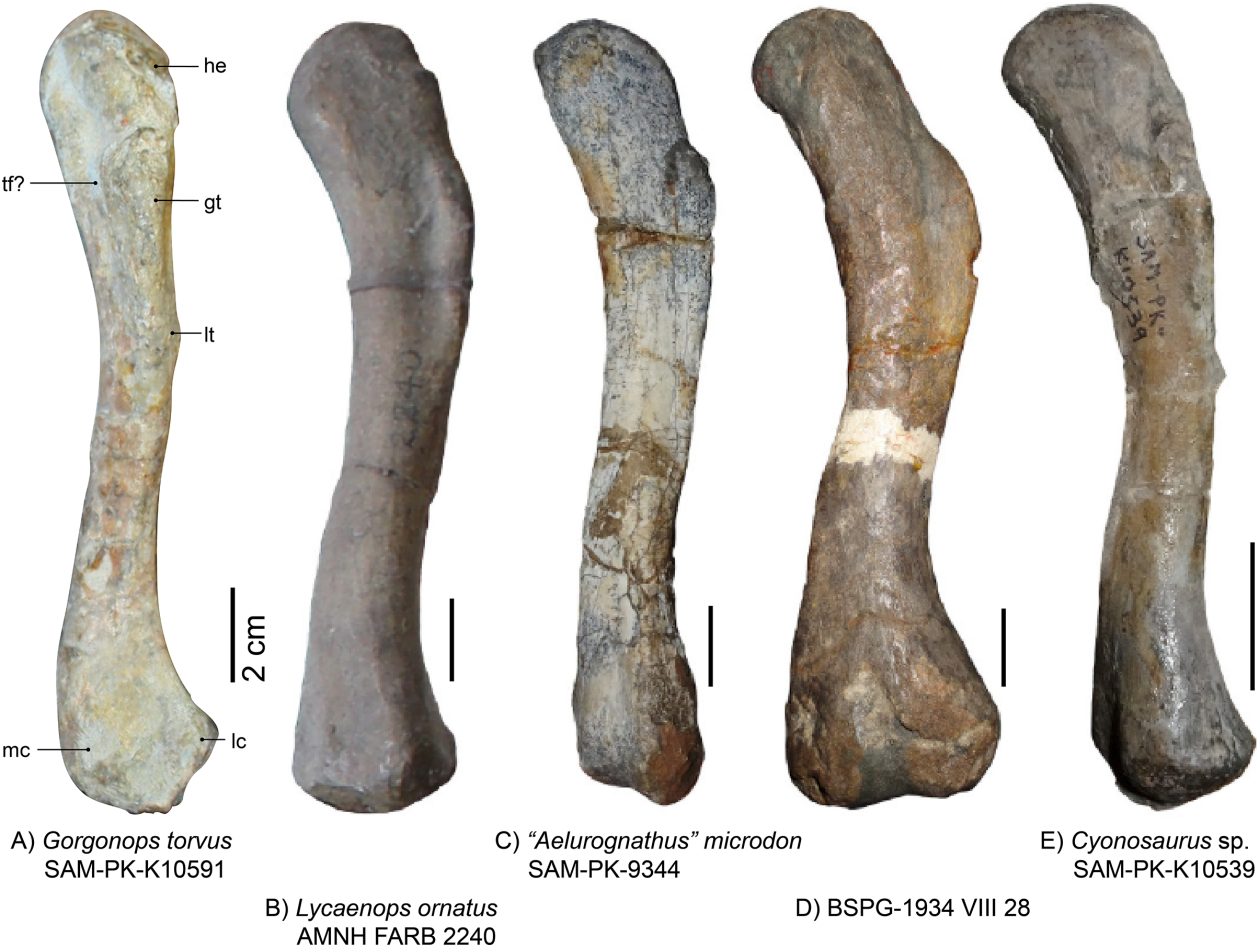
Photographs of gorgonopsian femora. (A) *Gorgonops torvus* (left, lateral view), (B) *Lycaenops ornatus* (left, dorsolateral view), (C) *“Aelurognathus” microdon* (left, ventrolateral view), (D), *“Scymnognathus”* sp. (left, dorsal view) and (E) *Cyonosaurus* sp. (left, lateral view). Scale bars = 2 cm. Abbreviations: gt, greater trochanter; he: head; lc, lateral condyle; lt, lesser trochanter, mc, medial condyle; tf?, trochlear fossa.

Comparison with other specimens is somewhat limited, as the preservation on the slab exposes mainly the lateral view of the bone, whereas specimens are usually depicted from ventral or dorsal in the literature. The right femoral head is not well preserved, but the left is very slim and not as expanded as in Colbert (1948)’s description of *Lycaenops* (AMNH FARB 2240, see also Fig. 12). The length of the femoral head is similar to that of other African gorgonopsians (*i.e*., a fifth of the length of the entire bone as in *e.g*., *Aelurognathus microdon*, SAM-PK-9344, or *Lycaenops ornatus*, AMNH FARB 2240, see Fig. 12). From the medial edge of the femoral head, a trench extends slightly diagonally towards the lateral edge and under the greater trochanter. This trench is deepest on the height of the proximal edge of the greater trochanter, possibly forming a trochanteric fossa. Laterally, the greater trochanter merges with the side of the femur and forms a lightly tapered crest. The distal condyles are separated dorsally by a weak depression; the medial condyle is slightly more bulbous. The distal epiphysis of the left femur is slimmer compared to other gorgonopsians (*i.e*., the more pronounced and bulbous condyles in UMZC T883), although the preservation of the condyles is not perfect. Overall, the femur morphology is most comparable to that of USNM 412381 (*Cyonosaurus*) and BSPG 1934 VIII 28 (Fig. 12), see Table S1. The curvature is more distinct in the USNM specimen (like that of another undescribed *Cyonosaurus* specimen, see Fig. 12), whereas it is slenderer than in the stockier *Suchogorgon golubevi* specimen PIN 4548/1 (although the width of this specimen has been exaggerated through crushing) or the similarly shaped “*Scymnognathus” parringtoni* (GPIT-PV-31579). The right femur, which appears more antero-posteriorly compressed, shows better-preserved condyles, but only the dorsal view is exposed (Fig. 11). Here, the shaft appears more robust and therefore the condyles are relatively slim. The insertion fossa for the musculus femoro-tibialis (Sigogneau-Russell, 1989) is weak, which is consistent with general gorgonopsian anatomy, apart from Colbert (1948)’s description of *Lycaenops*, in which he records ventrally ‘confluent’ condyles. At the position of the neck, the angle at which the femoral head protrudes from the shaft is ~150°. The dorsal surface of the shaft is smooth and featureless. Since both femora are so differently preserved in size and shape, and moreover the right limb bones are not in articulation with the rest of the skeleton, the possibility cannot be fully excluded that the right hindlimb pertains to another individual. Although this scenario is unlikely given their close association, only the left articulated limb bones are here considered in the revised diagnosis of *Gorgonops torvus*.

**Figure 13:**
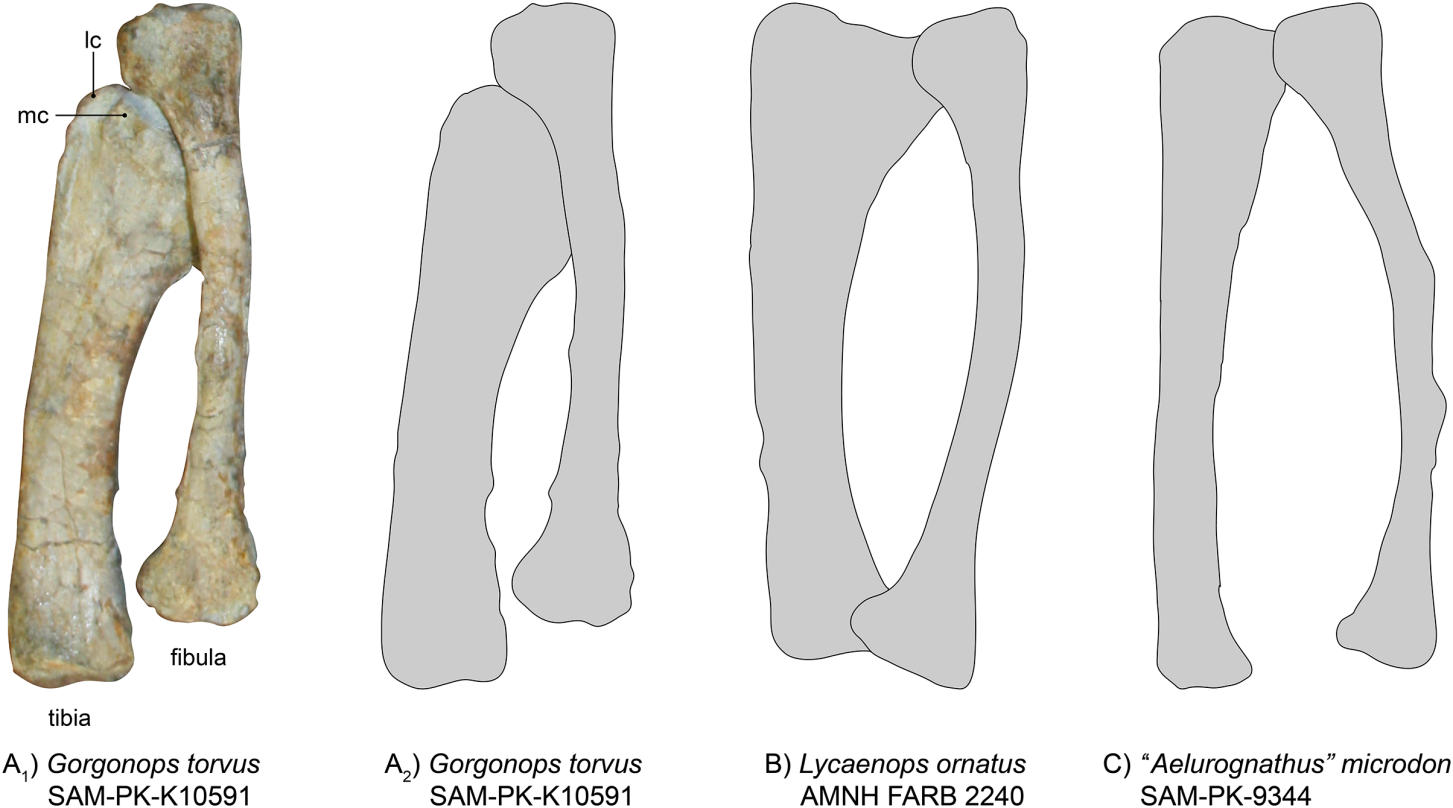
Comparison of various gorgonopsian tibiae and fibulae. (A_1_) Photograph of left tibia and fibula of *Gorgonops torvus*, (A_2_) outline drawing of left tibia and fibula of *Gorgonops torvus*, (B) outline drawing of left tibia and fibula of *Lycaenops ornatus* and (C) outline drawing of left tibia and fibula of “*Aelurognathus” microdon*. All in anterior view. Abbreviations: lc, lateral condyle; mc, medial condyle.

**Tibia-fibula complex**.—Both tibiae and fibulae are preserved (Fig. 13), but only the left ones are in articulation with femur and pes (Fig. 1), and thus provide a better reference for the general anatomy of *Gorgonops torvus*.

The right tibia is broken in half at about 1/3 of the length from the head. Of the right tibia, the anterior face is exposed; the same is true for the left tibia. The gorgonopsian tibia in general is a very robust bone (Colbert, 1948), shorter than the femur, and in SAM-PK-K10591 it is also much more massive than the fibula. The proximal condyle is large and complex, due to the contact surfaces for the femur as well as the more laterally oriented articulation surface for the fibula. A small trench is visible in both tibiae between the two articulating concavities for the femoral condyles. The medial of the two concave surfaces is much smaller. The lateral part of the proximal condyle also extends a little towards the fibula and medially. The shaft is quite robust and the distal end of the tibia is not very extended compared to the diaphysis (unlike in the slenderly built SAM-PK-9344, Fig. 13). The diaphysis almost entirely consists of a concave, saddle-like articulation surface for the astragalus. The entire bone is curved medially (similar to *Lycaenops ornatus*, see Fig. 13 (Colbert, 1948)).

The fibula typically bends outwards and is much slimmer than the tibia (Fig 13). The left, articulated fibula is slightly longer and much thinner than the tibia, especially concerning the diaphysis. The right fibula is partially preserved; only the proximal half is still present. A small bit of the distal condyle is preserved under the same part of the tibia.

Similar to concerns over the amount of flattening and distortion to the ulna-radius complex, the proportions of tibia and fibula (see Table 3) need to be treated with caution. SAM-PK-K10591 shows overall proportional similarities with *Lycaenops* and with BSPG 1934 VIII 28 (see Broili & Schröder, 1935). However, compared to the latter and *“Scymnognathus*” *parringtoni*, *Gorgonops* differs in the fibular width/length ratio, having an overall more slimly built fibula. As for its forelimb zeugopodial elements, the tibia-fibula complex of *Inostrancevia* appears to have been more robust than in the aforementioned taxa. The morphology of *Viatkogorgon* is very similar to the African taxa, however.

**Table 3:** Different ratios of tibia and fibula in various gorgonopsian specimens.

	SAM-PK-K10591, *Gorgonops torvus*	SAM-PK-9344, “*Aelurognathus*” *microdon*	AMNH FARB 2240, *Lycaenops ornatus*	BSPG 1934 VIII 28	GPIT-PV-31579, “*Scymnognathus*” *parringtoni*	PIN 2005/1758, *Inostrancevia alexandri*
Fibula head maximum width:tibia head maximum width	1:1.6	1:1.3	1:1.8	1:1.5	1:1.4	N/A
Tibia head maximum width:tibia length	1:3.1	1:4.8	1:3.1	1:3.5	1:3.1	1:2.4
Fibula head maximum width:fibula maximum length	1:5.6	1:5.9	1:5.8	1:5.1	1:4.3	N/A

**Figure 14:**
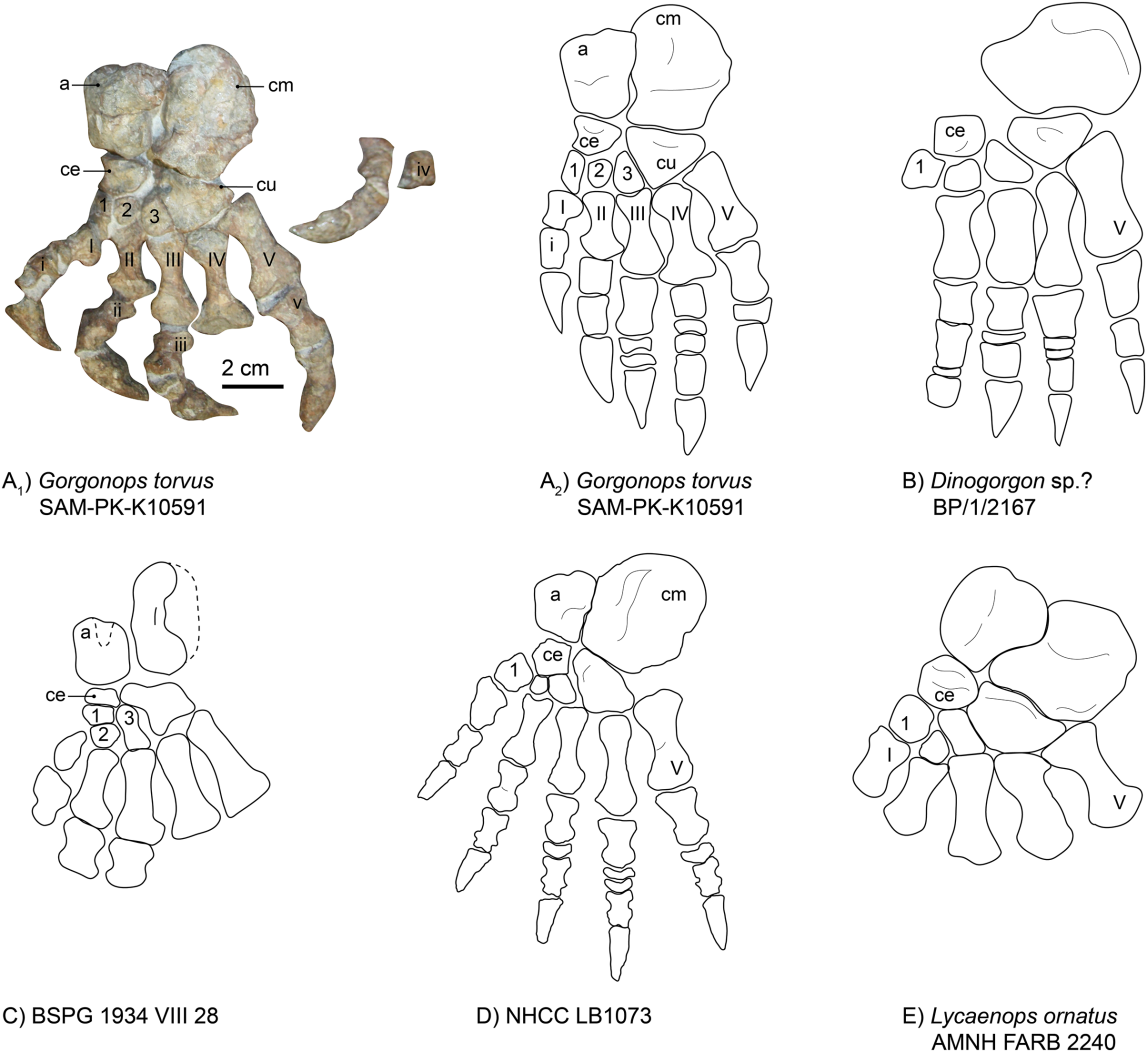
Comparison of various gorgonopsian pedes. (A) Photograph of left pes of *Gorgonops torvus*, (B) idealized drawing of left pes of *Gorgonops torvus*, (C) idealized drawing of left pes of *Dinogorgon* sp.?, (D) idealized drawing of left pes of BSPG 1934 VIII 28, (E) idealized drawing of pes of NHCC LB1073 (mirrored right pes) and (F) idealized drawing of left pes of *Lycaenops ornatus*. Scale bar = 2 cm. All in dorsal view. Abbreviations: a, astragalus; ce, centrale; cm, calcaneum; cu, cuboid. Arabic numerals indicate distal tarsal sequence and Roman numerals indicate metatarsal (capital) and phalangeal (lowercase) sequences.

**Pes**.—The left foot of SAM-PK-K10591 is completely preserved (Fig. 14A), which is a rarity for gorgonopsians. The phalangeal section of the fourth toe is disarticulated from the pes but closely associated. Due to its dorsal-up attitude of repose on the block, only the dorsal side of most elements can be observed. As is typical, the calcaneum is the largest element of the tarsus. It is almost double the area of the astragalus, but is generally flatter, irregular in shape with a rounded distal margin. It is longer proximodistally than wide mediolaterally. Medially, it is concave, accommodating the astragalus. On its dorsal side, it provides a large articulation surface for the fibula, where it is also widest overall (as in the Zambian gorgonopsian NHCC LB1073 recently described by Sidor (2022), see also Fig. 14D). Proximally, another flat surface provides a contact area for the merged fourth and fifth tarsalia (the cuboid). The triangular cuboid medially contacts the lateral (third) cuneiform, and laterally contacts the fourth and fifth metatarsals.

The gorgonopsian astragalus has been described as rectangular (Sigogneau-Russell, 1989), and this is true for SAM-PK-K10591 (see Fig. 14 and Table S1). The astragalus of *Lycaenops* is much more irregular in shape, with an especially bulbous contact with the calcaneum, and this element is not preserved in *Dinogorgon* (Figs. 14B and 14E). Additional astragali among published African specimens occur in BSPG 1934 VIII 28 (Fig. 14C) and NHCC LB1073 (Fig. 14D). Although it is not completely preserved in the former specimen, as a triangular piece in the middle of its proximal border is missing, it still appears to have been roughly rectangular, whereas it is more constricted along the medial midline in NHCC LB1073. As observed in other gorgonopsians (Colbert, 1948), the astragalus overlaps the calcaneum slightly proximally in SAM-PK-K10591. Like in NHCC LB1073 (Sidor, 2022), its contact surface for the tibia is rounded and a groove is present in the middle of the bone, but it is not as distinct. Its mediodistal edge therefore fits into the medial groove of the calcaneum. Distally, it contacts the centrale (or navicular), which then articulates with the other three tarsalia (lateral, middle, and medial cuneiforms). The centrale is much smaller than the cuboid and is, like the cuboid, triangular in shape. Its medial edge is concave, unlike in other gorgonopsians (see Fig 14). It has a straight articulation surface with the astragalus, another one laterally with the lateral cuneiform, and a further one medially with the middle and medial cuneiforms (like in NHCC LB1073 (Sidor, 2022)), which is not always the case for gorgonopsians. In *Lycaenops ornatus* (1948) the centrale was reconstructed as contacting only the first and third cuneiforms. More variations of this anatomical arrangement of tarsalia are present in other gorgonopsians, although these might be due to variable preservation and poor preparation (Sigogneau-Russell, 1989).

The medial cuneiform (first distal tarsal) is much slimmer than in the Zambian gorgonopsian NHCC LB1073 (Fig. 14D). Whereas it is the largest of the three in NHCC LB1073, the third (medial) cuneiform is largest in size in *Gorgonops* (Table S1). Both have a dorsally convex surface. Similar to NHCC LB1073, the second (middle) cuneiform is the smallest (also stated by Colbert (1948)), and although it does not overlap the second metatarsal like in the Zambian gorgonopsian, a concave bay is nonetheless present on the proximal surface of the second metacarpal. The third (lateral) cuneiform is somewhat trapezoidal (although its proximal contact surface with the centrale is very short) and has a large, tight articulation surface with the cuboid. The cuboid (proposed to be a fusion of the fourth and fifth cuneiforms (Hopson, 1995; Sigogneau, 1970; Sigogneau-Russell, 1989)) itself has a triangular shape, which is more pronounced compared to other gorgonopsian pedes. The shallower areas close to the contact with the calcaneum and on the edge with the third cuneiform resemble the condition present in NHCC LB1073. The latter fossa extends over the entire distal corner, which touches the third metatarsal, in *Gorgonops*, however.

Like the metacarpals, the fourth metatarsal of SAM-PK-K10591 is the longest (3.7 cm), followed by the fifth (3.2 cm). All metatarsals are long and slender and very constricted at the shaft (Fig. 14A). The second and fourth metatarsals are the most asymmetrical, with their distal ends being much wider than their proximal ends. The third metatarsal bears a proximal indentation for the articulation of the third cuneiform, similar to the second metatarsal in NHCC LB1073 (Sidor, 2022, and Fig. 14D). All three middle metatarsals have large bay-like features on the dorsal edge of their distal tip for contact with the phalanges. As in NHCC LB1073, the fifth metatarsal is much stockier than the fourth and is relatively wider at the articulation surface for the phalanx. The fifth metatarsals are flat in both SAM-PK-K10591 and NHCC LB1073 (Sidor, 2022).

As in NHCC LB1073, the proximal phalanges increase in overall robustness from the first to the fifth. The fourth proximal phalanx of the pes of *Gorgonops* is an exception, however, as it is much shorter than the third or the fifth. This is likely a preservational artifact and might not reflect the actual morphology. The Zambian gorgonopsian described by Sidor (2022) preserves a disc-like element in pedal digit V, as is also the case here.

The ungual phalanges of *Gorgonops* are typical for gorgonopsians as they are dorsally rounded, but they are more robust than in NHCC LB1073 (Sidor, 2022, and Fig. 14D) and more similar in proportions to those of *Dinogorgon* (Fig. 14B).

The pedal phalangeal formula is not known for many gorgonopsian specimens. It was previously only assumed for BP/1/2167 (*Dinogorgon rubidgei*), and more recently established for NHCC LB1073 (Sidor, 2022), see Table S1. In the past it has been assumed to be strictly the same as for the manus: **2-3-4-5-3** (Hopson, 1995). This also fits within the count hypothesized for *Viatkogorgon ivakhnenkoi*, although this was stated much more tentatively as “1 or 2-3-3 or 4-3 to 5-3” (Tatarinov, 2004). An exception to this pattern is the pedal phalangeal formula described by Sidor (2022) for NHCC LB1073, which presents a divergent pattern of 2-3-4-5-**4**, hinting at reductions in more derived groups such as rubidgeines, as also hypothesized by Hopson (1995).

A pedal phalangeal formula identical to the manual one of 2-3-4-5-3, would also be in concordance with reported ichnofossils of gorgonopsians. The ichnotaxon *Karoopes gansfonteinensis* was allocated to gorgonopsians (Marchetti et al., 2019) and the specimens are homopod tracks, *i.e*., same size and morphology of hand and foot (Leonardi, 1987). *Gorgonops torvus* follows this pattern, with two small disc-like phalangeal elements in the third and fourth toe. The complete right pes of NHCC LB1073 has only one disc-like element in the third toe and two in the fourth toe, however, whereas BP/1/2167 preserves a disc in its second toe. This morphological disparity might harbor diagnostic potential as more postcrania are described in the future.

## Discussion

### Taxonomic assignment of BSPG 1934 VIII 28 to *Gorgonops*

The taxonomic value of gorgonopsian postcranial material has historically been considered limited, but this is due in part to lack of detailed study and well-preserved comparative materials. Examination of SAM-PK-K10591 indicates that postcranial apomorphies, although they may be rare even in nearly complete skeletons, can nonetheless help resolve some uncertainties regarding taxonomic assignments. The Munich specimen BSPG 1934 VIII 28, originally described as *Scymnognathus* cf. *whaitsi* (Broili & Schröder, 1935), then later referred to *Gorgonops whaitsi* (Sigogneau, 1970) and subsequently Gorgonopsia gen. et sp. indet. (Gebauer, 2007) is one such taxonomic conundrum for which postcranial comparisons are essential.

The similar size and shared origin of BSPG 1934 VIII 28 and SAM-PK-K10591 in the *Endothiodon* AZ provide circumstantial evidence for possible conspecificity, but previously there was little morphological evidence for referral of the Munich specimen to *Gorgonops* (as noted by Gebauer (2007)). The weathered and poorly preserved skull of the specimen has been difficult to identify beyond an indeterminate gorgonopsian. However, examination of the postcranial anatomy of the Munich specimen compared to the herein described *Gorgonops torvus* specimen, SAM-PK-K10591, yielded significant similarities (see Table S1).

The anatomy of the pes (Fig. 14) provides several points of comparison. Although damaged in BSPG 1934 VIII 28, the astragalo-calcaneal proportions of this specimen more closely match that of SAM-PK-K10591 than other gorgonopsians for which these elements are known. The astragali in both specimens are large and rectangular in outline with calcanea longer than wide. This differs from the relatively smaller (in relation to the calcaneum) astragalus of the Zambian NHCC LB1073 or the much larger astragalus of *Lycaenops ornatus* (AMNH FARB 2240). The calcaneal proportions permit differentiation between the aforementioned two specimens as well as *Dinogorgon rubidgei* (BP/1/2167), in which the calcaneum is extremely broad. Morphology of the metatarsals is also comparable between BSPG 1934 VIII 28 and SAM-PK-K10591, with strongly constricted shafts unlike the broader, more robust elements in *Lycaenops* and *Dinogorgon*. Although the extreme rarity of pedes in Gorgonopsia necessarily limits our confidence in attributing species-level diagnostic value to the above characters, within the framework of known material we can at least state that these specimens are consistent with identification as a single taxon.

Furthermore, the ratio of the width of the head of both tibia and fibula to the length of the same bones are very similar in both specimens (see Table 3). Considering the femora (Fig. 12), the curvature, size and the weakness of the depression on the dorsal side of the distal condyles matches both specimens. In the ulna-radius complex, the proportions are slightly less informative, but the size of the insertion for the flexor muscles in the ulna are matching. Overall, all measurable characteristics preserved in both specimens match, whereas they differ from various other examined African gorgonopsian taxa (Table S1).

Lastly, even though the skull of BSPG 1934 VIII 28 is poor, as mentioned in earlier publications, a weak step-like ventrally directed bend in the maxilla, anterior to the canine, may still be identified. In conclusion, though we would reiterate certain caveats regarding recognition of autapomorphies and use of proportional characters when so few gorgonopsian species have any preserved postcrania, we believe the available evidence supports assigning the Munich specimen to *Gorgonops*, as previously proposed by Sigogneau (1970). Especially in comparison to other gorgonopsian postcrania from which BSPG 1934 VIII 28 differs in few (*e.g*., *Lycaenops*) or many (*e.g*., “*Scymnognathus*” *parringtoni*) aspects (Table S1), its similarity to SAM-PK-K10591 is striking and such differences that exist are readily interpretable as taphonomic. Given continued uncertainty as to the alpha taxonomy of *Gorgonops*, however, we refrain from assigning BSPG 1934 VIII 28 to a definitive species and determine it as *Gorgonops* sp.

### Postcranial disparity in Gorgonopsia

Gorgonopsian crania are generally considered morphologically conservative, and the same has been assumed for their postcrania, although the limited sample has hindered evaluation of this idea (Sigogneau-Russell, 1989). The number of gorgonopsian species represented by postcrania remains very limited, but given the broad phylogenetic scope of the taxa for which skeletal material is known (based on the phylogeny of Kammerer & Masyutin (2018)), it does seem to be the case that the clade as a whole had generally similar skeletons. At present, no consistent postcranial differences for diagnosing gorgonopsian subclades can be recognized. This does not mean that the gorgonopsian postcranium is devoid of phylogenetically informative characters, but identification of such will likely require better preserved materials, particularly for cranially well-diagnosed subclades (*e.g*., Rubidgeinae) for which postcranial data is minimal. At lower taxonomic levels, however, various aspects of postcranial morphology hold promise for diagnosis, with the potential for identification of isolated elements in the future.

The most autapomorphic gorgonopsian postcranium belongs to *Inostrancevia*. The scapula of this taxon is unmistakable, with a broad, plate-like blade unlike that of any other known gorgonopsian, but its zeugopodial anatomy is also unusual, with thickened radii and tibiae, particularly at their articular margins. Limited postcrania are known for the related Russian taxon *Sauroctonus progressus*, but its scapulocoracoid (preserved in PIN 156/5) is more similar in general proportions to those of African taxa, albeit with autapomorphic knob-like processes on the anterior and posterior margins of the scapular blade near its dorsal terminus. In the other member of the “Russian clade” (*sensu* Kammerer & Masyutin, 2018) preserving postcrania, *Suchogorgon golubevi*, the known elements are comparable to those of most African taxa, although the scapular blade (preserved in PIN 4548/1) is one of the narrowest known in Gorgonopsia. The wide variety of scapular morphologies among these closely related taxa refutes the idea that gorgonopsian skeletons were all completely homomorphic, despite extensive overlap in proportions (see Table S1). The Russian taxon with the most extensive postcranial record is *Viatkogorgon ivakhnenkoi*, in which the entire skeleton is known. The skeleton of this taxon is generally similar to African taxa (and *Sauroctonus* and *Suchogorgon*, excepting their aforementioned autapomorphic features). Although found in Russia, *Viatkogorgon ivakhnenkoi* has never been recovered as part of the “Russian clade” and is instead found as a basal gorgonopsian in phylogenetic analyses (Kammerer & Rubidge, 2022), so its shared features with “African clade” and “Russian clade” taxa likely represent symplesiomorphies.

Within the “African clade”, there is also clear variation in some postcranial elements. Scapular variation is observed between, *e.g*., “*Scymnognathus*” *parringtoni*, in which the dorsal edges of the blade flare out extremely widely anteroposteriorly (Gebauer, 2014), and *Arctognathus curvimola*, in which the blade is unusually short (Sigogneau-Russell, 1989). The new data provided by SAM-PK-K10591 indicate other postcranial elements showing either species-specific or otherwise taxonomically restricted distribution among African gorgonopsians (see Table S1). These include the shape of the radiale, which is triangular in *Gorgonops* and rubidgeines (at least those for which the manus is known, *e.g*., *Aelurognathus*), albeit with different, possibly diagnostic proportions between these taxa. Other variable features include the proportions of the unguals, which vary between short and stout in *Gorgonops* to long and blade-like in “*Scymnognathus*” *parringtoni*, potentially related to differences in prey capture between various gorgonopsian species, and the weakly pronounced ventral distinction between pubis and ischium in *Gorgonops*. Confirmation of this latter character requires additional, well-preserved material, as the extremely thin, laminar connection between ischium and pubis means that it is broken in many specimens, but proportional differences in more robust portions of the ischia of various African taxa indicate that some variation in pelvic characters within this group is real. Variation in phalangeal count, at least within the pes, appears to be present in African gorgonopsians (Sidor, 2022), potentially with multiple independent losses of the disc-like phalanges in different subclades. Phalangeal proportions also clearly vary among these taxa, likely also associated with aspects of lifestyle, as in the highly robust manus and pedes known in rubidgeines (seen in *Aelurognathus* and *Dinogorgon*).

Thus, although gorgonopsian postcrania are indeed similar at a broad comparative level, there is real variation in the group. As many gorgonopsian taxa co-occurred in the late Permian (Kammerer, 2016), such variation would have importance in filling distinct ecological niches with different functional morphologies (Lautenschlager et al., 2020). Postcranial trait variation is currently difficult to quantify, given that the elements involved are rarely completely preserved or taphonomically unaltered even in a single taxon, much less in multiple individuals of a range of taxa permitting statistically robust conclusions. Only by reporting more postcranial material of gorgonopsians can those shortcomings be overcome in the future. Hopefully, identification of postcranial apomorphies, such as those proposed here, will begin to permit referral of skull-less skeletal material to lower taxonomic levels, which could substantially increase the sample size for comparisons.

### Body mass estimation of *Gorgonops torvus*

**Figure 15:**
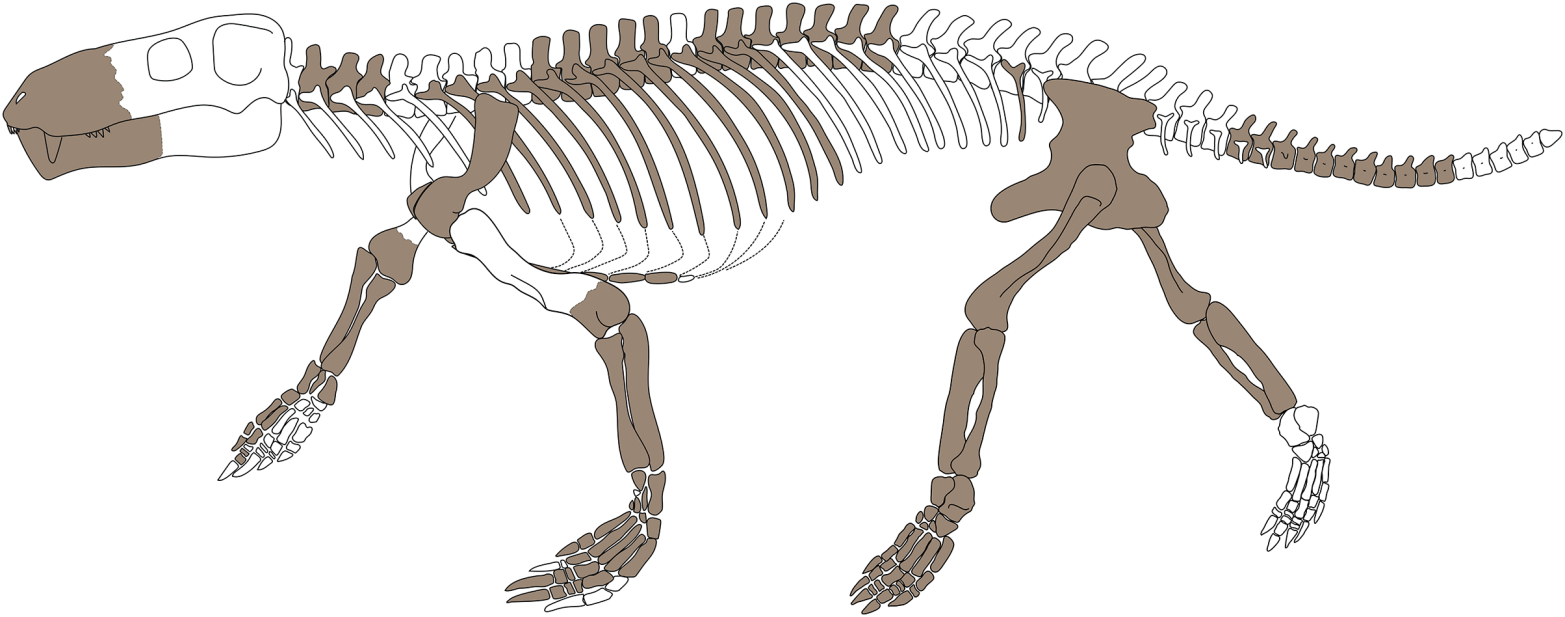
Reconstruction of the skeleton of *Gorgonops torvus* in walking pose, showing it from its left side. Based on the drawing of *Lycaenops ornatus* from Colbert (1948). Elements highlighted in brown are preserved elements of the specimen SAM-PK-K10591.

When reconstructing a fossil skeleton (Fig. 15), it may prove helpful to estimate the original body mass to more accurately determine its stance and agility (*e.g*., Benoit et al., 2017a; Orliac, Benoit & O’leary, 2012). Body mass can be an important factor when it comes to niche-partitioning (see below) so these estimates aid in the reconstruction of the lifestyle of an animal in its ecosystem (*e.g*., size of prey and hunter likely influence each other, according to Brocklehurst & Brink (2017)). Hopkins (2018) provided an overview of methods based on allometric relationships and correlations with extant taxa used to estimate the body weight in fossil specimens. Especially for synapsids however, Romano & Manucci (2019) have identified several caveats that may lead to an inaccurate estimates of the overall body mass. This is based on the fact that non-mammalian synapsid body morphology cannot be transferred easily to mammalian body morphology. Commonly, long bones are much stockier in the mammalian predecessors and their mode of life was different, therefore leading to different functional constraints to the bauplan.

Nevertheless, Benoit et al. (2017b) employed two additional methods to calculate body mass in dinocephalians (Body mass_1_ and Body mass_2_ below). Further, they used a method by Castanhinha et al. (2013), which is based on the method by Campione & Evans (2012, also mentioned in Hopkins (2018) and Romano & Manucci (2019) articles), who showed that the circumferences of humerus and femur should be given preference over the length of these elements (Body mass_3_ below).

Following are the estimated body masses for *Gorgonops torvus*, calculated with the three formulas in Benoit et al. (2017b):

**Body mass_1_** (in kg) = 2.7 * ((30)/10)^3^ = 72.9 kg (after Quiroga, 1980; Quiroga, 1984) (where 30 = estimated skull length in cm)

**Body mass_2_** (in kg) = 10^(3.13 * log10(300) − 5.59)^ = 145.68 kg (after Hu et al., 2005) (where 300 = estimated skull length in mm)

**Body mass_3_** (in kg) = 10^(2.749 * log10(12.1737) − 1.104)^ = 75.83 kg (after the general formula for quadrupedal tetrapods in Campione & Evans, 2012) (where 12.1737 = estimated femur circumference (calculated by using the widest (1.95 cm) + slimmest (1.65 cm) diameter of left femur) + estimated humerus circumference (calculated by using the estimated humeral diameter of the left (2 cm) and right (2.15 cm) humeri) in cm)

**Mean of Body mass_1−3_** = 98.17 kg

Whereas the values calculated for the Body mass_1_ and Body mass_3_ of *Gorgonops torvus* are very similar, they differ markedly from Body mass_2_. According to Benoit et al. (2017b), they might all be underestimating the actual body mass, especially when dealing with larger specimens with heavier bodies. Overall, the mean result is comparable with the skull length of 29.55 ± 0.88 cm (Yamaguchi et al., 2009) and associated body mass of 119.5–139.8 kg (Smuts, Robinson & Whyte, 1980) of a female lion (*Panthera leo*).

However, the results should be taken as an estimation, since extinct synapsids cannot simply be correlated in many instances to modern synapsids, even if they are part of an analogous ecological guild, such as hypercarnivores in this case. Furthermore, no formulae based on postcranial measurements have been developed for gorgonopsians in particular yet. The equations for the closest available taxonomic group, Dinocephalia, might not therefore accurately portray the body mass for Gorgonopsia, but there is enough overlap so that the applied equations can provide a tool for further interpreting physiological as well as ecological traits of gorgonopsians.

Generally, larger gorgonopsians such as *Gorgonops torvus* were likely at the top of the food chain. Through their body size, comparatively agile locomotion (Bendel et al., 2022), and powerful bites (Lautenschlager et al., 2020), they were able to fulfill the role of a top predator of late Permian terrestrial ecosystems.

### Gorgonopsians as ambush predators

Whenever contemporary animals live in the same area, it raises the question of how they partition limited available resources, especially amongst specialized predators. To reduce competition, related co-occurring taxa are often observed separating into somewhat different niches and guilds in an ecosystem:

Van Valkenburgh (1985) established five hunting types for large terrestrial predators, focusing on mammals. She listed ‘occasional/ambulatory’ for animals such as bears that only rarely hunt and ‘semifossorial’ for animals such as badgers that use digging in catching their prey. Furthermore, she included three more active categories: ‘ambush’, ‘pounce/pursuit’ and ‘pursuit’ hunting strategies, which gorgonopsians were more likely to have utilized. ‘Ambush’ is characterized as a stalking maneuver followed by a short burst of fast running and on capture, the forelimbs play a major role in killing. ‘Pounce/pursuit’ can be observed, *e.g*., by foxes that move long distances after which the hunt culminates in a quick pounce or chase. ‘Pursuit’ refers to long-range chases in which the forelimbs usually don’t have a critical function in holding down the prey during killing.

Anyonge (1996) further investigated the locomotion of fossil and extant carnivores. Although his study dealt exclusively with mammals, the potentially similar lifestyle of the therein analyzed carnivoran taxa may still permit use of these groups as tentative proxies for gorgonopsians, especially considering the lack of alternative studies with taxonomically closer groups. Anyonge used several indices for calculating the most likely function of skeletal elements during movement. Of those, only the crural index (maximum length of tibia = ~12.5 cm/maximum length of femur = ~16.5 cm) could be utilized when investigating the present *Gorgonops* specimen. The result of 0.758 falls between his values for ambulatory and ambush extant locomotor groups. He states that it is difficult to distinguish fossil taxa reliably between those two groups. We note here that the gorgonopsian value is far from the cursorial mean of 0.933 and very close to the crural index of *Thylacosmilus* (as applied in Argot, 2004). Further similarities to *Thylacosmilus* extend to the generally more robustly built forelimbs compared to more gracile hindlimbs, though in *Gorgonops* this may also have been related to its semi-erect posture, and associated gait intermediate between the typical sprawling ‘reptilian’ and the upright ‘mammalian’ gait.

The semi-erect and semiplantigrade gait (Fig. 15), paired with slender hindlimbs *vs*. robust forelimbs but a comparatively short tibia, as well as the interpreted gorgonopsian trackway ichnospecies *Karoopes gansfonteinensis*, suggests a relatively fast pace for gorgonopsians (Marchetti et al., 2019), which would in turn indicate an ‘ambush’ hunting strategy. This most likely excludes the ability to run fast over long distances, but allows a short chase and powerful grappling of the prey, followed by a strong bite (Lautenschlager et al., 2020) with the large, serrated canines. This is in keeping with the analysis of Cruickshank (1973), who made comparisons to the feeding mode of Komodo dragons, which use a slashing attack into the soft tissue of the stomach region to disable the prey, followed by a shaking movement of the head. To stabilize the body during this process, a wider stance is helpful. He additionally discussed the possibility that gorgonopsians fed on large-bodied and slow-moving animals such as large anomodonts (see also Fordyce, Chinsamy & Smith, 2012). This is in contrast to a recent biomechanical study of the skull, which points towards gorgonopsians overall possibly preying on smaller animals due to low jaw gape angles, but with a high possible bite force (Lautenschlager et al., 2020). These results do not necessarily exclude each other, however: the key messages suggest that gorgonopsians would have specialized on slower prey on which they inflicted severe wounds with their long canines. Smaller prey would have been easier to handle with a smaller gape angle, and the effective gape angle indeed would have varied due to different canine-lengths. However, if the belly region was being targeted, a small gape angle nevertheless would have sufficed to bring down a bigger animal.

Van Valkenburgh (1985) pointed out that it is common in recent guilds of predators that one guild dominates a habitat. This trend seems to have existed in late Permian landscapes as well, where gorgonopsian predators fulfilled this role. Gorgonopsians, with their overall conservative body type, must have competed within their clade for prey. However, occupying the same functional guild is not a hindrance to occupying different niches for food. Indeed, it has recently been shown that skulls of different gorgonopsian genera, too, show a large functional diversity although superficially appearing alike (Lautenschlager et al., 2020). Therefore, even while sharing the guild of ambush predators, gorgonopsians were able to compete successfully enough to diversify into different ecological niches during the middle and especially the late Permian.

This kind of niche-partitioning within the same guild is further supported by the new evidence of morphological variety within gorgonopsians presented in this study. Differences in size and proportions between contemporary taxa (*e.g*., *Aelurosaurus*, *Eriphostoma*, and *Gorgonops* in the *Endothiodon* AZ (Day & Smith, 2020)) would have provided the animals with varied opportunities for hunting. Although the paleoecological implications of the differences in pedal morphology between, *e.g*., *Gorgonops* and *Lycaenops*, or in scapular morphology between *Inostrancevia* and *Sauroctonus*, are not yet clear, we believe this represents a fruitful avenue for future, more explicitly functional studies, in association with primary descriptive work on new gorgonopsian postcranial materials.

## Conclusion

The postcranium of *Gorgonops torvus* illustrates a number of characters of taxonomic and functional importance. Although differences in postcranial morphology between taxa are currently minimal, there is some clear variation of at least lower-level taxonomic utility, although establishing characters of phylogenetic utility will require new, more extensive materials that more densely sample gorgonopsian subclades. For the time being, the cranial anatomy of gorgonopsians remains key to assigning specimens to broader taxonomic groups. The sparseness of postcranial elements collected with corresponding and properly identified skulls, in combination with less-than-ideal preservation, has limited more in-depth analyses and comparisons, particularly between closely related taxa. In the future, a larger quantity of postcranial descriptions of gorgonopsians would not only help resolve their systematics but also broaden our understanding of the evolution and lifestyles in this important group of synapsids.

## Supplemental Information

10.7717/peerj.15378/supp-1 Supplemental Information 1 Postcranial characters. Postcranial characters in gorgonopsians referred to in the main text

## Additional Information and Declarations

### Data Availability

The following information was supplied regarding data availability: The data are available in the descriptions in the article.
